# Temporal activation of WNT/β-catenin signaling is sufficient to inhibit SOX10 expression and block melanoma growth

**DOI:** 10.1038/s41388-020-1267-7

**Published:** 2020-04-01

**Authors:** Rexhep Uka, Christian Britschgi, Anja Krättli, Claudia Matter, Daniela Mihic, Michal J. Okoniewski, Marco Gualandi, Roger Stupp, Paolo Cinelli, Reinhard Dummer, Mitchell P. Levesque, Olga Shakhova

**Affiliations:** 1 Department of Medical Oncology and Hematology, University Hospital Zurich, University of Zurich, Wagistrasse 14, 8952 Schlieren, UK; 2 Department of Surgical Pathology, University Hospital Zurich, University of Zurich, Schmelzbergstrasse 12, 8091 Zurich, UK; 3 Scientific IT Services ETH Zurich, ETH Zurich, Weinbergstrasse 11, 8092 Zürich, UK; 4 Department of Trauma Surgery, University Hospital Zurich, University of Zurich, Sternwartstrasse 14, 8091 Zürich, UK; 5 Department of Dermatology, University Hospital Zurich, University of Zurich, Wagistrasse 14, 8952 Schlieren, UK; 6 Present Address: Ken and Ruth Davee Department of Neurology, Northwestern University Feinberg School of Medicine, Chicago, IL 60611 USA; 7 Present Address: The Lou and Jean Malnati Brain Tumor Institute, The Robert H. Lurie Comprehensive Cancer Center, Northwestern University Feinberg School of Medicine, Chicago, IL 60611 USA

**Keywords:** Cancer, Drug discovery

## Abstract

Despite advances in the systemic treatment of patients with metastatic melanoma using immune checkpoint and tyrosine kinase inhibitors (TKI), the majority of stage IV melanoma patients eventually succumb to the disease. We have previously identified the transcription factor Sox10 as a crucial player in melanoma, yet the underlying molecular mechanisms mediating Sox10-dependent tumorigenesis remain largely uncharacterized. Here, we show that MEK and RAF inhibitors do not suppress levels of SOX10 protein in patient-derived cells in vitro, as well as in melanoma patients in vivo. In a search for pharmacological inhibitors of SOX10, we performed a mass spectrometry-based screen in human melanoma cells. Subsequent analysis revealed that SOX10 directly interacts with β-catenin, which is a key mediator of canonical Wnt/β-catenin signaling. We demonstrate that inhibitors of glycogen synthase kinase 3 alpha/beta (GSK3α/β) efficiently abrogate SOX10 protein in human melanoma cells in vitro and in melanoma mouse models in vivo. The mechanism of action of GSK3-mediated SOX10 suppression is transcription-independent and relies on the presence of a proteasome degradable form of β-catenin. Taken together, we provide evidence that activation of canonical Wnt signaling has a profound effect on melanoma growth and is able to counteract Sox10-dependent melanoma maintenance both in vitro and in vivo.

## Introduction

Melanoma is the most aggressive type of skin cancer, characterized by highly invasive and metastatic features. Major risk factors are genetic predisposition and exposure to ultraviolet radiation, and the incidence of melanoma continues to increase worldwide [1]. While patients with early stage melanoma can often be cured by surgical intervention, patients with metastatic disease have a very poor prognosis. The historically high mortality rate was largely due to resistance of melanoma to conventional chemotherapy. Despite recent significant advances in systemic treatment of metastatic melanoma, including targeted therapies and immune checkpoint inhibitors, which might even lead to cure in some patients, metastatic melanoma remains a deadly disease for the majority, since they will eventually develop resistance to these approaches [2–7]. We have previously demonstrated that Sox10, a neural crest transcription factor, plays a crucial role in the development and maintenance of giant congenital melanocytic nevi and melanoma [8].

Melanoma originates from cells of the neural crest (NC) lineage and many parallels have been drawn between NC development and melanoma formation. During embryonic development, WNT signaling is implicated in neural crest induction and activation of WNT signaling stimulates the expression of Sox10 and other NC markers [9–11]. Conversely, blocking WNT signaling was shown to be associated with the inhibited expression of NC markers [12].

The canonical WNT pathway is highly controlled by a complex set of proteins, the so called destruction complex, to ensure the correct spatial and temporal expression of its target genes (reviewed in [13]). In the absence of Wnt ligands, the destruction complex consists among others of GSK3α/β, which keeps the levels of cytoplasmic β-catenin low. Once β-catenin is phosphorylated, it is subsequently ubiquitylated and ultimately degraded in the proteasome. Upon the binding of secreted Wnts to their cell surface receptors, β-catenin is no longer phosphorylated and thereby can escape the proteasome-mediated degradation. Finally, stabilized β-catenin translocates to the nucleus, where it associates with co-transcription factors to promote Wnt target gene transcription [14, 15].

The role of WNT signaling and the β-catenin pathway has been the subject of intensive research in the field of melanoma. However, its exact role has remained highly controversial to date. Canonical WNT signaling has been implicated in various aspects of melanoma pathogenesis, including proliferation and invasion [16]. Multiple studies have reported that elevated levels of β-catenin are associated with poor survival in patients and the overexpression of a stabilizing mutation of β-catenin in mice resulted in increased metastasis [17–20]. In contrast, several other reports have described that the activation of WNT signaling is beneficial for melanoma patients [21–24].

In our previous work, we have unambiguously demonstrated that Sox10 is indispensable for melanoma initiation and maintenance [8]. In this follow up study, we set out to identify agents to pharmacologically inhibit SOX10 protein in melanoma. An unbiased proteomics screen with the aim to identify proteins interacting with SOX10 revealed β-catenin as a SOX10 protein partner. Given the relationship between SOX10 and β-catenin during neural crest development, we manipulated WNT signaling in an attempt to regulate SOX10 expression. To our surprise and in contrast to the interaction in NC between Sox10 and β-catenin, activation of WNT signaling abolished SOX10 expression and inhibited melanoma formation. Three independent inhibitors of GSK3α/β (CHIR99021, LY2090314, and AZD1080) inhibited SOX10 protein levels in a panel of human melanoma cell cultures. Moreover, we show that the effect of GSK3α/β inhibition on SOX10 protein is β-catenin dependent. The data presented here demonstrate that the canonical WNT signaling is involved in fine-tuning SOX10 protein levels in melanoma. Our study is the first to demonstrate the protein-mediated regulation of the SOX10-WNT axis in melanoma biology, shedding light on the molecular mechanism of SOX10 regulation and resolving the controversy around the role of canonical WNT signaling in melanoma.

## Results

### Clinically used targeted BRAF or MEK inhibitors do not interfere with SOX10 expression levels

**Fig. 1:**
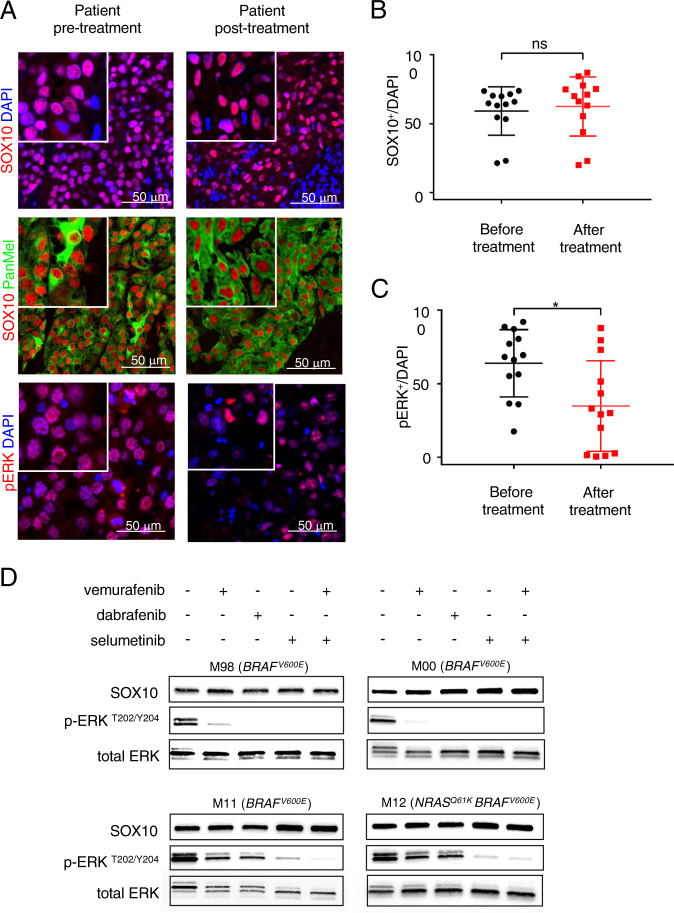
Clinically used targeted BRAF or MEK inhibitors do not interfere with SOX10 expression levels. **a** Representative images of immunohistochemical stainings of human melanoma patient derived biopsies (*n* = 13) before (left panel) and after (right panel) combined BRAF and MEK inhibitor treatment stained for SOX10 (red), pERK (red) and Pan Melanoma (PanMel), a cocktail of Melanoma antigen recognized by T cells-1 (MART-1) and Tyrosinase (green). Nuclei were counterstained with DAPI (blue). **b** Quantification of SOX10 expression levels pre- and post-BRAF and MEK inhibitor treatment. **c** Quantification of pERK expression levels pre- and post-BRAF and MEK inhibitor treatment. **d** Representative western blot for the indicated proteins in four different human melanoma cell lines. Levels of SOX10 expression are not decreased upon treatment with BRAF inhibitors (vemurafenib and dabrafenib, each 1 μM) or MEK inhibitor (selumetinib, 1 μM) after 24 h neither in single, nor in double treatments (vemurafenib and selumetinib). In MAPK inhibitor sensitive cell lines (M98, M00) pERK levels are not detectable anymore, which indicates the correct function of the drugs, whereas in resistant cell cultures (M11, M12) pERK levels were not affected (*n* = 3). In **b** and **c** data represent mean ± s.d. Statistical significance was determined by paired, two-tailed Student’s *t* test. *P < 0.05, **P < 0.01, ***P < 0.001 Western blots shown in **d** are representative. In each panel, *n* indicates the number of independent experiments performed.

We have previously shown that SOX10 is expressed in one hundred percent of primary human melanoma samples and that its expression is crucial for melanoma initiation, but also maintenance of established melanomas [8]. Several studies report that SOX10 expression rate is around 92–97% [25, 26]. Importantly, SOX10 is not only a melanoma marker but is functionally important for melanoma cells survival. Interestingly, one of the key downstream targets of SOX10, microphthalmia-associated transcription factor (MITF) has been previously linked to MAPK pathway [27]. Taken together, these observations prompted us to investigate the potential effect of current treatments on the expression of SOX10. Given the clinical observation that the majority of patients treated with small molecule inhibitors against BRAF or MEK kinases will eventually relapse (despite impressive initial responses), we investigated the capacity of those agents to suppress SOX10 expression in vivo and in vitro [2, 5, 6]. First, we performed immunohistochemistry for SOX10 in a set of human melanoma samples. These samples were derived from the same patients (*n* = 13) before and after the start of targeted therapy using a BRAF-inhibitor and MEK-inhibitor combination therapy (Fig. 1a; one representative patient is shown). Strikingly, there was no detectable difference in SOX10 expression after BRAFi treatment initiation in any of the paired biopsies (Fig. 1a, b). The efficiency of the treatment is demonstrated by the downregulation of pERK expression in the biopsies of the posttreatment patients (Fig. 1a, c).

Next, we performed western blot analysis in several early passage melanoma cultures derived from patient biopsies to analyze effects of MAPK-pathway suppression on SOX10 expression levels in vitro. We included both vemurafenib-sensitive and -resistant cultures (Supplementary Fig. S1a, b). SOX10 levels were not changed upon treatment with the BRAF inhibitors vemurafenib or dabrafenib, nor with the MEK1 inhibitor selumetinib after 24 h in vitro (Fig. 1d). Strikingly, even the combination of a BRAF and a MEK inhibitor, which is the new standard of care treatment in patients with BRAF^V600E^-mutated melanoma, did not alter SOX10 expressions levels [2, 5, 6]. Taken together, clinically used small molecule inhibitors of MAPK signaling, which are initially effective but eventually lead to secondary resistance, do not influence SOX10 expression levels, neither in vitro nor in vivo.

### β-catenin directly interacts with SOX10, and pharmacologic activation of WNT signaling suppresses SOX10 in a β-catenin-dependent manner

**Fig. 2:**
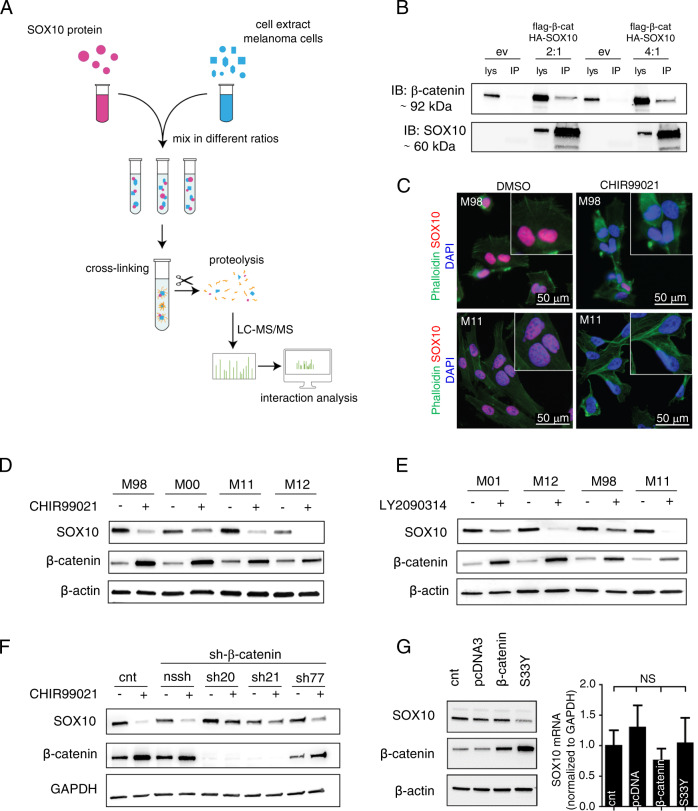
β-catenin directly interacts with SOX10, and pharmacological activation of Wnt signaling suppresses SOX10 in a β-catenin-dependent manner. **a** Schematic representation showing the nLc Orbitrap mass spectrometry (MS) analysis to identify SOX10-interacting proteins. M010817 whole cell extract (WCE) was analyzed performing nLc Orbitrap MS at CovalX. **b** Interaction of SOX10 and β-catenin was confirmed by co-immunoprecipitation using an anti-HA antibody for the pull down. **c** Representative immunocytochemical stainings indicating the subcellular localization of SOX10 (in red) and actin cytoskeleton (in green) in the MAPK inhibitor sensitive (M98, upper panel) and in the resistant human melanoma cell line M11 (lower panel) in presence of DMSO (left panel) or CHIR99021 (for 24 h, 6 μM) (right panel). Inserts show higher magnification of SOX10 immunostainings (*n* = 3). **d** A representative western blot of the indicated proteins in presence or absence of the GSK3 α/β inhibitor CHIR99021 (for 24 h at 6 μM) in patient-derived melanoma cell cultures sensitive (M98, M00) against targeted MAPK inhibitors or resistant (M11, M12) (*n* = 5). **e** A representative western blot for the indicated proteins in presence or absence of the GSK3 α/β inhibitor LY2090314 (for 24 h at 100 nM) in patient-derived melanoma cell cultures sensitive (M01 and M98) or resistant against MAPK inhibitors (M12 and M11) (*n* = 3). **f** A representative western blot for the indicated proteins of M11 human melanoma cell culture stably expressing *shCTNNB1* constructs cultured in presence or absence of CHIR99021 for 24 h, 6 μM (*n* = 3). **g** On the left, representative western blot of the indicated proteins in the MAPK inhibitor resistant human melanoma cell culture M11 transiently expressing wild type β-catenin or a stabilizing mutated form of β-catenin (S33Y). Empty pCDNA3 was used as control (cnt). β-actin was used as loading control. On the right, *SOX10* mRNA expression levels normalized to *GAPDH* in the MAPK inhibitor resistant human melanoma cell culture M11 transiently expressing wild type β-catenin or a stabilized mutated form of β-catenin (S33Y) (*n* = 3). Data represent mean ± s.d. In **d** and **e**, β-actin was used as loading control. In **f** and **g**, GAPDH was used as loading control. In each panel, *n* indicates the number of independent experiments performed.

Because tyrosine kinase inhibition did not alter SOX10 levels and given the fact that transcription factors are notoriously difficult to target therapeutically, we aimed to find new protein–protein interaction partners of SOX10 to identify novel approaches to regulate its function. To this end, we performed interaction mass spectrometry (MS) using whole cell extracts (WCE) isolated from the M010817 primary melanoma culture mixed with recombinant SOX10 protein in different ratios. The mixtures were cross-linked and analyzed using LC-MS/MS (Fig. 2a) [28]. This highly stringent approach identified β-catenin as a potential interactor of SOX10 (Supplementary Fig. S2a, b).

To confirm the MS-predicted protein–protein interaction between SOX10 and β-catenin, we performed a co-immunoprecipitation (Co-IP) assay (Fig. 2b). We overexpressed flag-tagged β-catenin and HA-tagged SOX10 in different ratios in HEK293T/17 cells (which are SOX10-negative), and analyzed HA-immunoprecipitate for expression of β-catenin using SDS-PAGE and western blotting. This approach confirmed the interaction of SOX10 and β-catenin on the protein level, as indicated by the detected β-catenin signal in the Co-IP eluates.

In order to interfere only with the signaling function of β-catenin without compromising cell–cell adhesion, we inhibited the negative upstream regulator, namely, GSK3α/β, a component of the β-catenin destruction complex. We used CHIR99021 (hereafter called either CHIR99021 or CHIR), a small chemical compound that competes with ATP for the binding site in GSK3α/β and thereby inhibits GSK3α/β, leading to the activation of WNT signaling.

As shown in Fig. 2c, immunocytochemical analysis demonstrated that treatment with CHIR99021 strongly diminished SOX10 protein expression levels in our panel of primary melanoma cultures. Vehicle control treated cells showed a strong nuclear SOX10 expression, whereas 24 h after CHIR99021 treatment the nuclei were SOX10 negative (Fig. 2c). The suppression of SOX10 protein expression was also evident in western blot experiments on WCE (Fig. 2d). Importantly, this striking effect on SOX10 protein levels was present in all melanoma cultures, irrespective of whether they harbor activating BRAF p.V600E, NRAS p.Q61K, or both mutations, and irrespective of whether they are sensitive to MAPK inhibition or resistant (Supplementary Fig. S1a). To exclude off-target effects of CHIR99021, we made use of two additional GSK3α/β inhibitors (LY2090314 and AZD1080), which showed essentially the same effects (Fig. 2e and Supplementary Fig. S3 and S4).

To test whether SOX10 downregulation is dependent on the presence of β-catenin, we generated cell cultures stably expressing control sh (nssh), which represents a real hairpin but not targeting any human gene and shRNAs targeting the *CTNNB1* gene transcript, which encodes β-catenin. As a consequence of β-catenin knockdown in M111031 melanoma cultures, the suppression of SOX10 protein expression upon CHIR99021 treatment was fully rescued (Fig. 2f). We also transfected M111031 melanoma cultures with a plasmid encoding either wild type β-catenin or a stabilized mutant p.S33Y that cannot be phosphorylated by GSK3α/β and thus not degraded by the proteasome. When expressing β-catenin p.S33Y, there was a clear downregulation of SOX10 protein (Fig. 2g). This is a further indication that suppression of SOX10 by CHIR99021 is mediated by β-catenin stabilization, and is not a direct effect of GSK3α/β inhibition. Taken together, our findings suggest that CHIR99021-mediated downregulation of SOX10 is β-catenin dependent. These findings point to a new mechanism for the regulation of SOX10 expression via activation of WNT/β-catenin signaling.

### Constitutively active WNT signaling due to β-catenin stabilizing mutations does not repress SOX10 expression levels and wild type β-catenin is predominantly located in the cytoplasm in melanoma biopsies

**Fig. 3:**
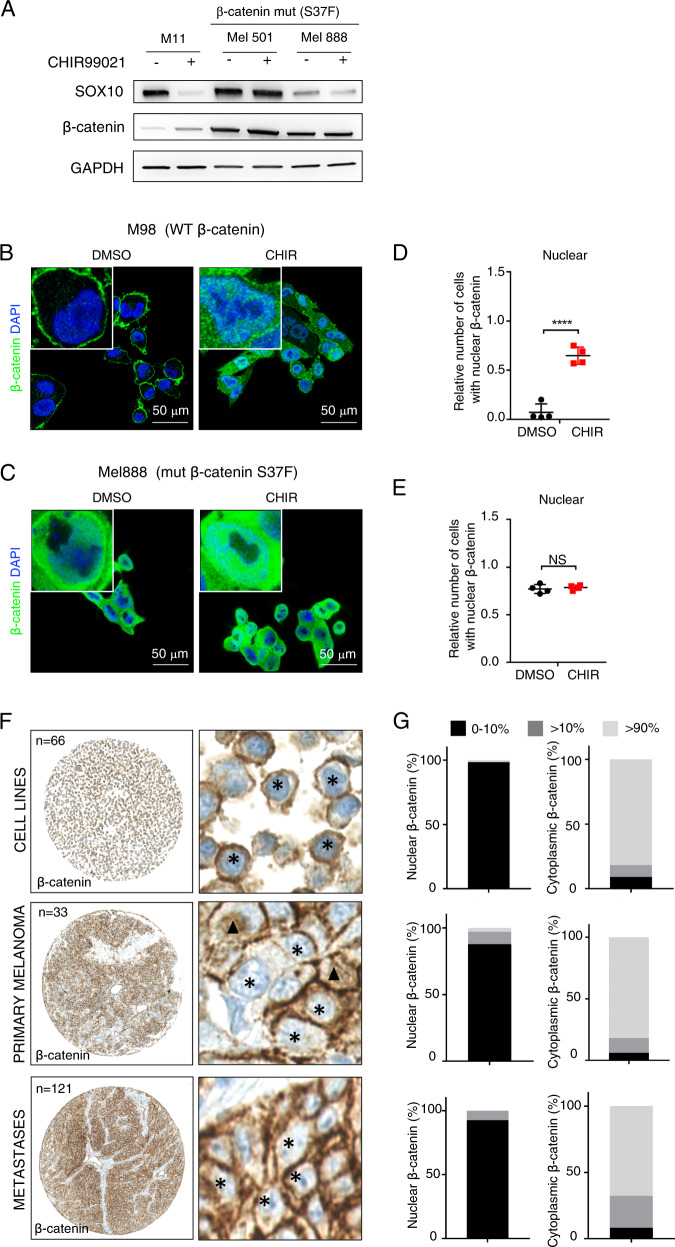
Wild type β-catenin translocates into the nucleus upon CHIR99021-mediated stabilization in melanoma cell cultures. **a** A representative western blot for the indicated proteins of cell lines carrying a stabilizing mutation in β-catenin (p.S37F) (Mel501, Mel888). M11 expresses wild type β-catenin and serves as a control. The human melanoma-derived cell lines were cultured in presence or absence of CHIR99021 for 24 h, 6 μM (*n* = 3). **b** Representative immunocytochemical stainings indicating the subcellular localization of β-catenin (in green) in a melanoma patient-derived cell culture carrying wild type β-catenin (M98) in presence of DMSO (left panel) or 6 µM CHIR99021 (right panel) for 24 h. Inserts show higher magnification of immunostainings. **c** Representative immunocytochemical stainings indicating the subcellular localization of β-catenin (in green) in a melanoma cell line harboring the stabilizing p.S37F mutation in β-catenin (Mel888) in presence of DMSO (left panel) or 6 µM CHIR99021 (right panel) for 24 h. Inserts show higher magnification of immunostainings. **d** β-catenin nuclear localization analysis of M98 upon DMSO or 24 h of 6 µM CHIR99021 treatment, respectively. **e** β-catenin nuclear localization analysis of Mel888 upon DMSO or 24 h of 6 µM CHIR99021 treatment, respectively. **f** Tissue Micro Array (TMA) of melanoma cell lines (upper panel) *n* = 66, primary melanoma (middle panel) *n* = 33, or metastases (lower panel) *n* = 121, showing the cytoplasmic localization of β-catenin. The TMAs were analyzed according to the expression level of β-catenin both nuclear and cytoplasmic. The TMAs were so divided into nuclear (N) and cytoplasmic (C) localization of β-catenin. The samples were divided into three groups with expression in <10%, (Cell lines: *N* = 98.5% *C* = 9,1%; Primary melanoma: *N* = 87.9%, *C* = 6.1%; Metastases: *N* = 92.6%, *C* = 8.3%) between 10% and 90% (Cell lines: *N* = 0%, *C* = 9,1%; Primary melanoma: *N* = 9,1% C = 12.1%; Metastases: *N* = 7.4% *C* = 24%) and more than 90% (Cell lines: *N* = 1.5%, *C* = 81.8%; Primary melanoma: *N* = 3.0% C = 81,8%; Metastases: *N* = 0% *C* = 67.8%). Asterisks indicate β-catenin negative nuclei, filled triangles β-catenin positive ones. **g** The quantification is shown in the respective analyzed tissues. Data represent mean ± s.d. Statistical significance was determined by unpaired, two-tailed Student’s *t* test. *P < 0.05, **P < 0.01, ***P < 0.001 In each panel, *n* indicates the number of independent experiments performed.

All the melanoma cultures from our panel express relatively low levels of β-catenin and we therefore included two more cell lines that harbor the stabilizing β-catenin exon 3 p.S37F mutation, Mel888 and Mel501 (Fig. 3a and Supplementary Fig. S5) [29]. Strikingly, treatment with CHIR99021 did not further increase β-catenin levels in mutant cell lines, nor reduce SOX10 protein levels. This suggests that SOX10 is regulated by acute changes of β-catenin stability, but not by continued expression of its constitutively stabilized mutant forms, which are retained in the nucleus. This is in line with immunocytochemical experiments, which illustrate that wild type β-catenin is mostly retained at the cell membrane in melanoma culture M980513 and translocates to the nucleus upon stabilization induced by CHIR99021 treatment (Fig. 3b, d). β-catenin p.S37F, however, is strongly expressed throughout the cytoplasm and the nucleus and does not change upon GSK3α/β inhibition in Mel888 (Fig. 3c, e). Moreover, CHIR99021 only shows an effect on SOX10 expression levels when WNT signaling is not constitutively hyper-activated by β-catenin mutations.

Constitutive activation of canonical WNT signaling (e.g., by stabilizing β-catenin exon 3 mutations) has been implicated in different types of cancer. There is an ongoing debate about the impact of the subcellular localization of β-catenin on melanomagenesis. It has been shown that the majority of benign nevi are positive for nuclear β-catenin, whereas melanoma progression is accompanied by the loss of nuclear β-catenin expression [23, 30–32]. We complemented those published observations by performing an analysis of data from The Cancer Genome Atlas (TCGA) project [33]. Of 469 (31/469 6.61%) melanoma cases deposited in the TCGA database, only 6.61% carried a β-catenin mutation, of which 43.2% were located in exon 3 (Supplementary Fig. S6 and Table 1).

**Table 1:** Mutation analysis of the CTNNB1gene in melanoma patients.

Study	Sample ID	Cancer type	Protein change	Annotation	Functional impact	Mutation type	Copy #	COSMIC	MS	VS	Center	Chromosome	Start Pos	End Pos	Ref	Var	Allele Freq (T)	Allele Freq (N)	Variant reads	Ref reads	Variant reads (N)	Ref reads (N)	# Mut in sample
Skin cutaneous melanoma (TCGA, PanCancer Atlas)	TCGA-D9-A6EG-06	Cutaneous melanoma	S45P	OncoKB: Likely Oncogenic, level NA;CIViC: Diagnostic: 2, Prognostic: 1;MyCancerGenome: present;CancerHotspot: yes;3DHotspot: no	MutationAssessor: impact: medium, score: 2.215;SIFT: impact: deleterious, score: 0;Polyphen-2: impact: probably_damaging, score: 0.939	Missense_Mutation		817	–	–	–	3	41266136	41266136	T	C			24	55	0	66	65
Skin cutaneous melanoma (TCGA, Provisional)	TCGA-D9-A6EG-06	Cutaneous melanoma	S45P	OncoKB: Likely Oncogenic, level NA;CIViC: Diagnostic: 2, Prognostic: 1;MyCancerGenome: present;CancerHotspot: yes;3DHotspot: no	MutationAssessor: impact: medium, score: 2.215;SIFT: impact: deleterious, score: 0;Polyphen-2: impact: probably_damaging, score: 0.939	Missense_Mutation		817	Somatic	NA	broad.mit.edu	3	41266136	41266136	T	C				52	−1	−1	74
Skin cutaneous melanoma (TCGA, PanCancer Atlas)	TCGA-ER-A19O-06	Cutaneous melanoma	T41A	OncoKB: Oncogenic, level NA;CIViC: Predictive: 3, Diagnostic: 2, Prognostic: 1;MyCancerGenome: not present;CancerHotspot: yes;3DHotspot: no	MutationAssessor: impact: medium, score: 2.68;SIFT: impact: deleterious, score: 0;Polyphen-2: impact: possibly_damaging, score: 0.844	Missense_Mutation		941	–	–	–	3	41266124	41266124	A	G			7	14	0	43	213
Skin cutaneous melanoma (TCGA, Provisional)	TCGA-ER-A19O-06	Cutaneous melanoma	T41A	OncoKB: Oncogenic, level NA;CIViC: Predictive: 3, Diagnostic: 2, Prognostic: 1;MyCancerGenome: not present;CancerHotspot: yes;3DHotspot: no	MutationAssessor: impact: medium, score: 2.68;SIFT: impact: deleterious, score: 0;Polyphen-2: impact: possibly_damaging, score: 0.844	Missense_Mutation		941	Somatic	NA	broad.mit.edu	3	41266124	41266124	A	G				14	−1	−1	155
Skin cutaneous melanoma (TCGA, PanCancer Atlas)	TCGA-WE-A8ZN-06	Cutaneous melanoma	S37F	OncoKB: Likely Oncogenic, level NA;CIViC: NA;MyCancerGenome: present;CancerHotspot: yes;3DHotspot: no	MutationAssessor: impact: medium, score: 2.66;SIFT: impact: deleterious, score: 0;Polyphen-2: impact: probably_damaging, score: 0.962	Missense_Mutation		493	–	–	–	3	41266113	41266113	C	T			17	34	0	38	135
Skin cutaneous melanoma (TCGA, PanCancer Atlas)	TCGA-FS-A1YY-06	Cutaneous melanoma	S45C	OncoKB: Likely Oncogenic, level NA;CIViC: NA;MyCancerGenome: present;CancerHotspot: yes;3DHotspot: no	MutationAssessor: impact: medium, score: 2.565;SIFT: impact: deleterious, score: 0;Polyphen-2: impact: probably_damaging, score: 0.968	Missense_Mutation		817	–	–	–	3	41266137	41266137	C	G			15	27	0	54	165
Skin cutaneous melanoma (TCGA, Provisional)	TCGA-FS-A1YY-06	Cutaneous melanoma	S45C	OncoKB: Likely Oncogenic, level NA;CIViC: NA;MyCancerGenome: present;CancerHotspot: yes;3DHotspot: no	MutationAssessor: impact: medium, score: 2.565;SIFT: impact: deleterious, score: 0;Polyphen-2: impact: probably_damaging, score: 0.968	Missense_Mutation		817	Somatic	NA	broad.mit.edu	3	41266137	41266137	C	G				26	−1	−1	169
Skin cutaneous melanoma (TCGA, PanCancer Atlas)	TCGA-D3-A8GV-06	Cutaneous melanoma	S45del	OncoKB: Likely Oncogenic, level NA;CIViC: NA;MyCancerGenome: present;CancerHotspot: yes;3DHotspot: no		In_Frame_Del		57	–	–	–	3	41266134	41266136	CTT	–			24	32	0	57	339
Skin cutaneous melanoma (TCGA, PanCancer Atlas)	TCGA-EE-A2M6-06	Cutaneous melanoma	S45del	OncoKB: Likely Oncogenic, level NA;CIViC: NA;MyCancerGenome: present;CancerHotspot: yes;3DHotspot: no		In_Frame_Del		57	–	–	–	3	41266134	41266136	CTT	–			15	19	0	41	571
Skin cutaneous melanoma (TCGA, Provisional)	TCGA-EE-A2M6-06	Cutaneous melanoma	S45del	OncoKB: Likely Oncogenic, level NA;CIViC: NA;MyCancerGenome: present;CancerHotspot: yes;3DHotspot: no		In_Frame_Del		57	Somatic	NA	broad.mit.edu	3	41266134	41266136	CTT	–				20	−1	−1	460
Skin cutaneous melanoma (TCGA, PanCancer Atlas)	TCGA-FS-A4F9-06	Cutaneous melanoma	G34R	OncoKB: Likely Oncogenic, level NA;CIViC: NA;MyCancerGenome: not present;CancerHotspot: yes;3DHotspot: no	MutationAssessor: impact: medium, score: 2.66;SIFT: impact: deleterious, score: 0;Polyphen-2: impact: probably_damaging, score: 0.997	Missense_Mutation		285	–	–	–	3	41266103	41266103	G	A			19	18	0	51	372
Skin cutaneous melanoma (TCGA, Provisional)	TCGA-FS-A4F9-06	Cutaneous melanoma	G34R	OncoKB: Likely Oncogenic, level NA;CIViC: NA;MyCancerGenome: not present;CancerHotspot: yes;3DHotspot: no	MutationAssessor: impact: medium, score: 2.66;SIFT: impact: deleterious, score: 0;Polyphen-2: impact: probably_damaging, score: 0.997	Missense_Mutation		285	Somatic	NA	broad.mit.edu	3	41266103	41266103	G	A				18	−1	−1	378
Skin cutaneous melanoma (TCGA, PanCancer Atlas)	TCGA-EB-A5SH-06	Cutaneous melanoma	T41N	OncoKB: Likely Oncogenic, level NA;CIViC: NA;MyCancerGenome: not present;CancerHotspot: yes;3DHotspot: no	MutationAssessor: impact: medium, score: 2.335;SIFT: impact: deleterious, score: 0.03;Polyphen-2: impact: probably_damaging, score: 0.95	Missense_Mutation		941	–	–	–	3	41266125	41266125	C	A			14	39	0	45	88
Skin cutaneous melanoma (TCGA, Provisional)	TCGA-EB-A5SH-06	Cutaneous melanoma	T41N	OncoKB: Likely Oncogenic, level NA;CIViC: NA;MyCancerGenome: not present;CancerHotspot: yes;3DHotspot: no	MutationAssessor: impact: medium, score: 2.335;SIFT: impact: deleterious, score: 0.03;Polyphen-2: impact: probably_damaging, score: 0.95	Missense_Mutation		941	Somatic	NA	broad.mit.edu	3	41266125	41266125	C	A				39	−1	−1	97
Skin cutaneous melanoma (TCGA, PanCancer Atlas)	TCGA-D9-A149-06	Cutaneous melanoma	T41I	OncoKB: Likely Oncogenic, level NA;CIViC: NA;MyCancerGenome: not present;CancerHotspot: yes;3DHotspot: no	MutationAssessor: impact: medium, score: 2.68;SIFT: impact: deleterious, score: 0;Polyphen-2: impact: probably_damaging, score: 0.931	Missense_Mutation		941	–	–	–	3	41266125	41266125	C	T			12	26	0	57	710
Skin cutaneous melanoma (TCGA, Provisional)	TCGA-D9-A149-06	Cutaneous melanoma	T41I	OncoKB: Likely Oncogenic, level NA;CIViC: NA;MyCancerGenome: not present;CancerHotspot: yes;3DHotspot: no	MutationAssessor: impact: medium, score: 2.68;SIFT: impact: deleterious, score: 0;Polyphen-2: impact: probably_damaging, score: 0.931	Missense_Mutation		941	Somatic	NA	broad.mit.edu	3	41266125	41266125	C	T				25	−1	−1	202
Skin cutaneous melanoma (TCGA, PanCancer Atlas)	TCGA-D3-A8GS-06	Cutaneous melanoma	W383G	OncoKB: Likely Oncogenic, level NA;CIViC: NA;MyCancerGenome: not present;CancerHotspot: yes;3DHotspot: no	MutationAssessor: impact: medium, score: 3.02;SIFT: impact: deleterious, score: 0;Polyphen-2: impact: possibly_damaging, score: 0.745	Missense_Mutation		6	–	–	–	3	41274897	41274897	T	G			13	112	0	110	172
Skin cutaneous melanoma (TCGA, PanCancer Atlas)	TCGA-EE-A180-06	Cutaneous melanoma	K292E	OncoKB: Predicted Oncogenic, level NA;CIViC: NA;MyCancerGenome: not present;CancerHotspot: yes;3DHotspot: no	MutationAssessor: impact: medium, score: 2.81;SIFT: impact: deleterious, score: 0;Polyphen-2: impact: probably_damaging, score: 0.912	Missense_Mutation		2	–	–	–	3	41267290	41267290	A	G			22	48	0	61	439
Skin cutaneous melanoma (TCGA, Provisional)	TCGA-EE-A180-06	Cutaneous melanoma	K292E	OncoKB: Predicted Oncogenic, level NA;CIViC: NA;MyCancerGenome: not present;CancerHotspot: yes;3DHotspot: no	MutationAssessor: impact: medium, score: 2.81;SIFT: impact: deleterious, score: 0;Polyphen-2: impact: probably_damaging, score: 0.912	Missense_Mutation		2	Somatic	NA	broad.mit.edu	3	41267290	41267290	A	G				39	−1	−1	458
Skin cutaneous melanoma (TCGA, PanCancer Atlas)	TCGA-EE-A181-06	Cutaneous melanoma	P714L	OncoKB: Unknown, level NA;CIViC: NA;MyCancerGenome: not present;CancerHotspot: no;3DHotspot: no	MutationAssessor: impact: medium, score: 2.285;SIFT: impact: tolerated_low_confidence, score: 0.07;Polyphen-2: impact: benign, score: 0.075	Missense_Mutation		1	–	–	–	3	41280628	41280628	C	T			12	32	0	80	4469
Skin cutaneous melanoma (TCGA, Provisional)	TCGA-EE-A181-06	Cutaneous melanoma	P714L	OncoKB: Unknown, level NA;CIViC: NA;MyCancerGenome: not present;CancerHotspot: no;3DHotspot: no	MutationAssessor: impact: medium, score: 2.285;SIFT: impact: tolerated_low_confidence, score: 0.07;Polyphen-2: impact: benign, score: 0.075	Missense_Mutation		1	Somatic	NA	broad.mit.edu	3	41280628	41280628	C	T				29	−1	−1	2503
Skin cutaneous melanoma (TCGA, PanCancer Atlas)	TCGA-D3-A1Q5-06	Cutaneous melanoma	C429G	OncoKB: Unknown, level NA;CIViC: NA;MyCancerGenome: not present;CancerHotspot: no;3DHotspot: no	MutationAssessor: impact: medium, score: 2.94;SIFT: impact: deleterious, score: 0;Polyphen-2: impact: probably_damaging, score: 0.994	Missense_Mutation		2	–	–	–	3	41275119	41275119	T	G			35	40	0	95	295
Skin cutaneous melanoma (TCGA, Provisional)	TCGA-D3-A1Q5-06	Cutaneous melanoma	C429G	OncoKB: Unknown, level NA;CIViC: NA;MyCancerGenome: not present;CancerHotspot: no;3DHotspot: no	MutationAssessor: impact: medium, score: 2.94;SIFT: impact: deleterious, score: 0;Polyphen-2: impact: probably_damaging, score: 0.994	Missense_Mutation		2	Somatic	NA	broad.mit.edu	3	41275119	41275119	T	G				39	−1	−1	306
Skin cutaneous melanoma (TCGA, PanCancer Atlas)	TCGA-D3-A2JN-06	Cutaneous melanoma	P687S	OncoKB: Unknown, level NA;CIViC: NA;MyCancerGenome: not present;CancerHotspot: no;3DHotspot: no	MutationAssessor: impact: low, score: 1.59;SIFT: impact: tolerated, score: 0.32;Polyphen-2: impact: benign, score: 0	Missense_Mutation			–	–	–	3	41278183	41278183	C	T			34	87	0	99	721
Skin cutaneous melanoma (TCGA, Provisional)	TCGA-D3-A2JN-06	Cutaneous melanoma	P687S	OncoKB: Unknown, level NA;CIViC: NA;MyCancerGenome: not present;CancerHotspot: no;3DHotspot: no	MutationAssessor: impact: low, score: 1.59;SIFT: impact: tolerated, score: 0.32;Polyphen-2: impact: benign, score: 0	Missense_Mutation			Somatic	NA	broad.mit.edu	3	41278183	41278183	C	T				80	−1	−1	292
Skin cutaneous melanoma (TCGA, PanCancer Atlas)	TCGA-D3-A2JN-06	Cutaneous melanoma	P687L	OncoKB: Unknown, level NA;CIViC: NA;MyCancerGenome: not present;CancerHotspot: no;3DHotspot: no	MutationAssessor: impact: medium, score: 2.14;SIFT: impact: deleterious, score: 0.03;Polyphen-2: impact: benign, score: 0.066	Missense_Mutation			–	–	–	3	41278184	41278184	C	T			34	89	0	100	721
Skin cutaneous melanoma (TCGA, Provisional)	TCGA-D3-A2JN-06	Cutaneous melanoma	P687L	OncoKB: Unknown, level NA;CIViC: NA;MyCancerGenome: not present;CancerHotspot: no;3DHotspot: no	MutationAssessor: impact: medium, score: 2.14;SIFT: impact: deleterious, score: 0.03;Polyphen-2: impact: benign, score: 0.066	Missense_Mutation			Somatic	NA	broad.mit.edu	3	41278184	41278184	C	T				81	−1	−1	292
Skin cutaneous melanoma (TCGA, PanCancer Atlas)	TCGA-EE-A3AC-06	Cutaneous melanoma	Q545*	OncoKB: Unknown, level NA;CIViC: NA;MyCancerGenome: not present;CancerHotspot: no;3DHotspot: no		Nonsense_Mutation		1	–	–	–	3	41275738	41275738	C	T			18	27	0	80	1834
Skin cutaneous melanoma (TCGA, Provisional)	TCGA-EE-A3AC-06	Cutaneous melanoma	Q545*	OncoKB: Unknown, level NA;CIViC: NA;MyCancerGenome: not present;CancerHotspot: no;3DHotspot: no		Nonsense_Mutation		1	Somatic	NA	broad.mit.edu	3	41275738	41275738	C	T				25	−1	−1	844
Skin cutaneous melanoma (TCGA, PanCancer Atlas)	TCGA-D9-A6E9-06	Cutaneous melanoma	D390E	OncoKB: Unknown, level NA;CIViC: NA;MyCancerGenome: not present;CancerHotspot: no;3DHotspot: no	MutationAssessor: impact: medium, score: 3.12;SIFT: impact: deleterious, score: 0;Polyphen-2: impact: probably_damaging, score: 0.982	Missense_Mutation			–	–	–	3	41274920	41274920	T	G			18	31	0	55	155
Skin cutaneous melanoma (TCGA, Provisional)	TCGA-D9-A6E9-06	Cutaneous melanoma	D390E	OncoKB: Unknown, level NA;CIViC: NA;MyCancerGenome: not present;CancerHotspot: no;3DHotspot: no	MutationAssessor: impact: medium, score: 3.12;SIFT: impact: deleterious, score: 0;Polyphen-2: impact: probably_damaging, score: 0.982	Missense_Mutation			Somatic	NA	broad.mit.edu	3	41274920	41274920	T	G				31	−1	−1	168
Skin cutaneous melanoma (TCGA, PanCancer Atlas)	TCGA-D9-A6EC-06	Cutaneous melanoma	L762P	OncoKB: Unknown, level NA;CIViC: NA;MyCancerGenome: not present;CancerHotspot: no;3DHotspot: no	MutationAssessor: impact: low, score: 1.215;SIFT: impact: tolerated_low_confidence, score: 0.14;Polyphen-2: impact: benign, score: 0.003	Missense_Mutation			–	–	–	3	41280772	41280772	T	C			10	27	0	45	3621
Skin cutaneous melanoma (TCGA, Provisional)	TCGA-D9-A6EC-06	Cutaneous melanoma	L762P	OncoKB: Unknown, level NA;CIViC: NA;MyCancerGenome: not present;CancerHotspot: no;3DHotspot: no	MutationAssessor: impact: low, score: 1.215;SIFT: impact: tolerated_low_confidence, score: 0.14;Polyphen-2: impact: benign, score: 0.003	Missense_Mutation			Somatic	NA	broad.mit.edu	3	41280772	41280772	T	C				26	−1	−1	3905
Skin cutaneous melanoma (TCGA, PanCancer Atlas)	TCGA-EE-A29A-06	Cutaneous melanoma	P606L	OncoKB: Unknown, level NA;CIViC: NA;MyCancerGenome: not present;CancerHotspot: no;3DHotspot: no	MutationAssessor: impact: low, score: 1.7;SIFT: impact: tolerated, score: 0.11;Polyphen-2: impact: benign, score: 0.408	Missense_Mutation		1	–	–	–	3	41277853	41277853	C	T			68	95	0	214	210
Skin cutaneous melanoma (TCGA, Provisional)	TCGA-EE-A29A-06	Cutaneous melanoma	P606L	OncoKB: Unknown, level NA;CIViC: NA;MyCancerGenome: not present;CancerHotspot: no;3DHotspot: no	MutationAssessor: impact: low, score: 1.7;SIFT: impact: tolerated, score: 0.11;Polyphen-2: impact: benign, score: 0.408	Missense_Mutation		1	Somatic	NA	broad.mit.edu	3	41277853	41277853	C	T				88	−1	−1	215
Skin cutaneous melanoma (TCGA, PanCancer Atlas)	TCGA-D3-A2JC-06	Cutaneous melanoma	P100H	OncoKB: Unknown, level NA;CIViC: NA;MyCancerGenome: not present;CancerHotspot: no;3DHotspot: no	MutationAssessor: impact: medium, score: 2.735;SIFT: impact: deleterious, score: 0;Polyphen-2: impact: probably_damaging, score: 0.987	Missense_Mutation			–	–	–	3	41266502	41266502	C	A			4	24	0	56	3091
Skin cutaneous melanoma (TCGA, PanCancer Atlas)	TCGA-D3-A8GI-06	Cutaneous melanoma	T595I	OncoKB: Unknown, level NA;CIViC: NA;MyCancerGenome: not present;CancerHotspot: no;3DHotspot: no	MutationAssessor: impact: low, score: 1.15;SIFT: impact: tolerated, score: 0.43;Polyphen-2: impact: benign, score: 0.393	Missense_Mutation			–	–	–	3	41277315	41277315	C	T			58	104	0	148	4217
Skin cutaneous melanoma (TCGA, PanCancer Atlas)	TCGA-EE-A20F-06	Cutaneous melanoma	R93S	OncoKB: Unknown, level NA;CIViC: NA;MyCancerGenome: not present;CancerHotspot: no;3DHotspot: no	MutationAssessor: impact: medium, score: 2.69;SIFT: impact: deleterious, score: 0.03;Polyphen-2: impact: possibly_damaging, score: 0.677	Missense_Mutation			–	–	–	3	41266482	41266482	G	T			6	57	0	179	1819
Skin Cutaneous Melanoma (TCGA, PanCancer Atlas)	TCGA-EE-A29P-06	Cutaneous melanoma	E55*	OncoKB: Unknown, level NA;CIViC: NA;MyCancerGenome: not present;CancerHotspot: no;3DHotspot: no		Nonsense_Mutation			–	–	–	3	41266166	41266166	G	T			3	33	0	84	409
Skin cutaneous melanoma (TCGA, PanCancer Atlas)	TCGA-ER-A19J-06	Cutaneous melanoma	L46M	OncoKB: Unknown, level NA;CIViC: NA;MyCancerGenome: not present;CancerHotspot: no;3DHotspot: no	MutationAssessor: impact: low, score: 1.665;SIFT: impact: tolerated, score: 0.09;Polyphen-2: impact: possibly_damaging, score: 0.635	Missense_Mutation		4	–	–	–	3	41266139	41266139	C	A			3	32	0	53	182
Skin cutaneous melanoma (TCGA, PanCancer Atlas)	TCGA-GN-A262-06	Cutaneous melanoma	X509_splice	OncoKB: Unknown, level NA;CIViC: NA;MyCancerGenome: not present;CancerHotspot: no;3DHotspot: no		Splice_Site			–	–	–	3	41275629	41275629	G	T			3	33	0	68	949
Skin cutaneous melanoma (TCGA, PanCancer Atlas)	TCGA-GN-A26D-06	Cutaneous melanoma	M398I	OncoKB: Unknown, level NA;CIViC: NA;MyCancerGenome: not present;CancerHotspot: no;3DHotspot: no	MutationAssessor: impact: low, score: 0.935;SIFT: impact: tolerated, score: 0.2;Polyphen-2: impact: benign, score: 0.003	Missense_Mutation			–	–	–	3	41275028	41275028	G	T			4	34	1	97	618
Skin cutaneous melanoma (TCGA, PanCancer Atlas)	TCGA-W3-AA1V-06	Cutaneous melanoma	A532V	OncoKB: Unknown, level NA;CIViC: NA;MyCancerGenome: not present;CancerHotspot: no;3DHotspot: no	MutationAssessor: impact: low, score: 0.975;SIFT: impact: tolerated, score: 0.68;Polyphen-2: impact: benign, score: 0.01	Missense_Mutation			–	–	–	3	41275700	41275700	C	T			7	43	0	47	4472
Skin cutaneous melanoma (TCGA, Provisional)	TCGA-EE-A182-06	Cutaneous melanoma	W25*	OncoKB: Unknown, level NA;CIViC: NA;MyCancerGenome: not present;CancerHotspot: no;3DHotspot: no		Nonsense_Mutation		3	Somatic	NA	broad.mit.edu	3	41266077	41266077	G	A				23	−1	−1	1198

As a read-out for constitutive WNT signaling activation in patients, we next analyzed whether β-catenin was retained in the nucleus in a cohort of human melanoma samples. To this end, we performed immunohistochemical staining of a tissue micro array (TMA) containing human samples from primary melanoma (*n* = 33), metastases (*n* = 121), and melanoma cultures (*n* = 66). The samples were categorized according to SOX10 positivity and the presence and subcellular localization of β-catenin. As expected and previously published, all the cores stained positive for nuclear SOX10 expression (data not shown) [8]. With respect to β-catenin, we found that a high number of human samples present low nuclear expression in primary cases (87.9%), metastases (92.6%), and primary melanoma cultures (98.5%) (Fig. 3f, g).

Taken together, we can conclude that β-catenin is wild type and expressed in the cytoplasm in the majority of melanoma biopsies and therefore, the primary melanoma cultures used in our in vitro experiments represent a reliable system to analyze the WNT signaling state of melanomas in patients.

### Activation of WNT/β-catenin signaling reduces invasion and proliferation in melanoma cells in vitro

**Fig. 4:**
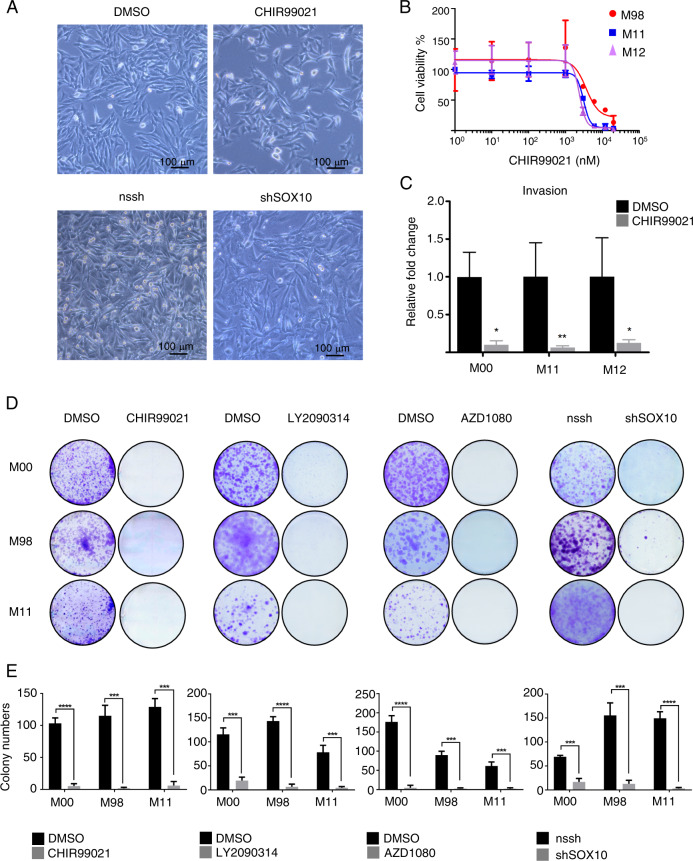
Activation of Wnt/β-catenin signaling reduces proliferation and invasion in melanoma cells in vitro. **a** A representative picture of the MAPK inhibitor-resistant melanoma patient-derived cell culture M11 in presence of DMSO (left side) or CHIR99021 (6 µM) for 24 h (right side) in the upper panel. In the bottom panel a representative picture of nssh (left side) and shSOX10 (right side) expressing M11 cell culture. **b** Relative inhibition of proliferation of M98 (sensitive to MAPK inhibitor), M11, and M12 (resistant) melanoma cell cultures after treatment with increasing concentrations of CHIR99021 at 72 h. Drug concentration is indicated in a logarithmic scale (*n* = 3). **c** Relative fold changes in invasion in vitro of MAPK inhibitor sensitive (M00) and resistant (M11, M12) human melanoma cell cultures treated with 6 µM CHIR99021 are shown compared with DMSO (ctrl) (*n* = 3). **d** Representative images of colony formation assay of MAPK inhibitor sensitive cell cultures (M00 and M98) and resistant cell culture (M11) in presence of 6 µM CHIR99021, 100 nM LY2090314, 10 µM AZD1080, and shSOX10-expressing, respectively (*n* = 3). **e** Relative quantification of colony formation assay. Data represent mean ± s.d. Statistical significance was determined by unpaired, two-tailed Student’s *t* test. *P < 0.05, **P < 0.01, ***P < 0.001. In each panel, *n* indicates the number of independent experiments performed.

Melanoma cells depend on SOX10 for survival, and SOX10 haploinsufficiency prevents or even reverts melanoma formation [8]. We therefore assessed next the phenotypic consequences of reducing SOX10 protein levels by activating WNT signaling in melanoma cells in vitro. Proliferation was reduced by 100% in long-term proliferation assays. Importantly, these findings could be reliably confirmed using three different GSK3 α/β inhibitors (CHIR99021, LY2090314, AZD1080) and shSOX10 constructs (Fig. 4d, e). Moreover, in MTT assays performed after 72 h of CHIR99021 treatment, with IC50 values of 3087, 3642, and 2386 nM in M111031, M980513, and M121224 cells showed an inhibition of proliferation of 100% (Fig. 4b). These observations underline the specific effect of WNT activation and hence SOX10 depletion in reducing proliferation of melanoma cultures, rather than an off target effect of CHIR99021. Of note, CHIR99021 was equally effective in vemurafenib-sensitive or -resistant cultures. Moreover, cellular invasion was also significantly reduced in all the tested primary melanoma cultures after only 24 h of CHIR99021 treatment (Fig. 4c). Importantly, the cells were still largely alive after 24 h of CHIR99021 treatment illustrating that the observed reduction in invading cells is not only a reflection of a greatly reduced absolute cell number (Fig. 4a).

Several lines of evidence implicate a function of the WNT pathway in cancer. In particular, mutations in β-catenin leading to constitutively activated WNT signaling are observed in different types of cancer, most prominently in colorectal cancer [23]. Moreover, WNT was identified to be an oncogene in breast cancer. Therefore, constitutive activation of WNT signaling is oncogenic in most contexts. To ensure that CHIR99021 treatment does not constitutively activate WNT signaling, we analyzed how long β-catenin expression is elevated upon a pulse of CHIR99021. As early as 48 h after changing to control medium, we detected a full recovery to the endogenous β-catenin and SOX10 protein expression levels in both TKI-sensitive and -resistant melanoma cell lines (Supplementary Fig. S7).

### Suppression of SOX10 by pharmacologic activation of WNT signaling is not transcriptional, but mediated by the proteasome

It was previously shown that Sox10 can act as nuclear-cytoplasmic shuttle protein [34]. This, together with the fact that upon activation of Wnt signaling, β-catenin translocates into the nucleus, raises two hypotheses: (i) since both SOX10 and β-catenin are transcription factors and thus can exert their functions partially in the nucleus, the CHIR99021-mediated regulation of SOX10 occurs in the nucleus, potentially at a transcriptional level. (ii) Alternatively, taking into account that SOX10 is an active shuttle protein and β-catenin is degraded via the proteasome, SOX10 degradation might involve the cytoplasmic proteasome machinery.

**Fig. 5:**
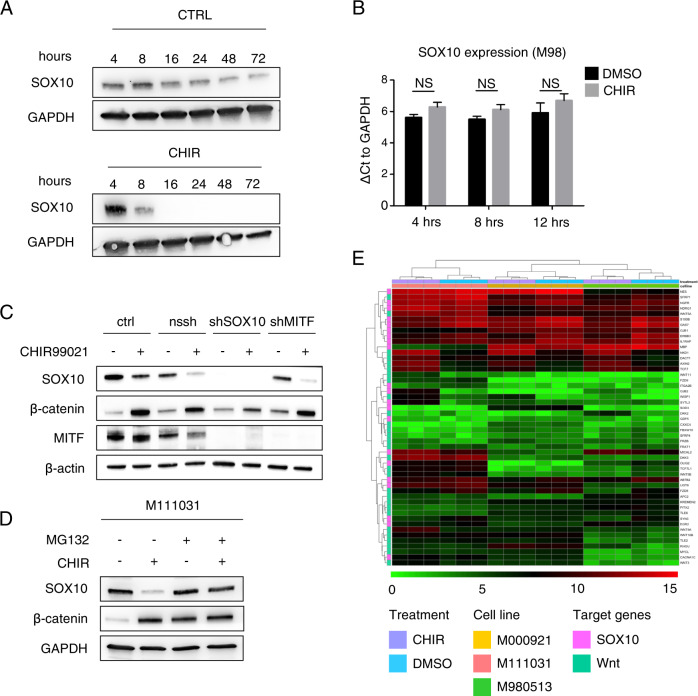
Suppression of SOX10 by pharmacologic activation of WNT signaling is not transcriptional, but mediated by the proteasome. **a** Representative western blots of the indicated proteins in the MAPK inhibitor resistant human melanoma cell culture M12, at different timepoints of CHIR99021 (6 μM) treatment (timepoints are shown directly in the figure). GAPDH was used as loading control (*n* = 3). **b** RNA levels in MAPK inhibitor sensitive (M98) human melanoma cell culture (ΔCt to *GAPDH* expression) after 4, 8, and 12 h of CHIR99021 (6 µM) treatment are shown compared with DMSO (ctrl) (*n* = 3). **c** Representative western blot of the indicated proteins in the MAPK inhibitor sensitive human melanoma cell culture (M98). A representative western blot for the indicated proteins of M98 human melanoma cell culture stably expressing *shSOX10* or *shMITF* constructs cultured in presence or absence of CHIR99021 for 24 h, 6 μM. Nssh represents the scrambled shRNA used as control construct (*n* = 3). **d** Representative western blot of the indicated proteins in presence of CHIR99021 (6 μM) and/or MG-132 (20 μM) (in single or double treatments) for 16 h. GAPDH was used as a loading control (*n* = 3). **e** Heatmap demonstrating the gene expression of SOX10 and Wnt target genes of M98 and M00 (MAPK inhibitor sensitive cell cultures) and M11 (resistant to MAPK inhibitor) in either presence or absence of CHIR99021. Data on the heat map represent log2 of sequencing reads assigned to genes. Statistical significance was determined by unpaired, two-tailed Student’s *t* test in **b**. *P < 0.05, **P < 0.01, ***P < 0.001. In each panel, *n* indicates the number of independent experiments performed.

To test the first hypothesis, we characterized changes in *SOX10* mRNA levels in the melanoma cultures upon CHIR99021 treatment. As shown in Fig. 5b, no significant difference was detected in mRNA levels in CHIR99021 treated cells as compared with controls for up to 12 h after addition of CHIR99021 to the cells (Fig. 5b and Supplementary Fig. S8a, b). Notably, SOX10 protein levels started to be downregulated as early as 8 h after addition of CHIR99021 (Fig. 5a). In line with these findings, an acute stabilization of β-catenin by transient overexpression of β-catenin p.S33Y in M111031 showed similar effects. SOX10 protein levels were suppressed, whereas *SOX10* mRNA levels remained unchanged (Fig. 2g). In addition to SOX10, its downstream target MITF plays a crucial role in neural crest cells that underwent differentiation into the melanocyte lineage [15, 35–37]. MITF is known as the “master transcription regulator” of the melanocyte lineage inducing expression of a plethora of other genes. Moreover, it has been reported that Wnt/β-catenin positively regulates MITF expression [20, 38]. In line with this notion we sought to determine, whether the effect of CHIR99021 on SOX10 was mediated via MITF. To this end, we generated a cell line stably expressing *shMITF*, in which MITF knockdown was readily detectable. CHIR99021-mediated SOX10 downregulation was detected also in absence of MITF, indicating that the Wnt activation-mediated effect on SOX10 is independent of MITF (Fig. 5c). Taken together, these data suggest that CHIR99021-mediated downregulation of SOX10 is exerted either at the translational level or is mediated by a change in SOX10 protein stability. To assess this further, we next cultured the melanoma cells in the presence or absence of the proteasome inhibitor MG132 (Fig. 5d). As expected, proteasome inhibition stabilized β-catenin, since it is rapidly degraded by the proteasome in the absence of WNT signaling (reviewed in [13]). However, SOX10 was not suppressed as a consequence of this MG132-mediated stabilization, nor by the combination of MG132 and CHIR99021. In fact, the CHIR99021-mediated SOX10 suppression was fully rescued by proteasomal inhibition. This strongly suggests that the mechanism of reduction of SOX10 protein levels following pharmacologic, acute β-catenin stabilization is mediated via proteasomal degradation.

### Activation of WNT/β-catenin signaling modulates the expression of SOX10 transcriptional targets

We investigated transcriptional changes in melanoma cultures upon CHIR99021 treatment. For this purpose, we used three different melanoma cultures, two of which are sensitive to MAPK inhibitors used in clinical practice (M980513 and M000921), and one derived from a patient cell line that is intrinsically resistant to vemurafenib (M111031). We compared the expression changes of SOX10 and Wnt target genes between cells cultured in the presence or absence of CHIR99021 for 24 h in triplicates. Efficiency of CHIR99021 treatment is illustrated in Supplementary Figure 8. Twenty-four hours after treatment start, we extracted total RNA and performed a genome wide transcriptional profiling using RNA sequencing. Hierarchical clustering of SOX10 and Wnt transcriptional target genes indicates that treatment with CHIR99021 has a major impact on the expression of SOX10 target genes (Fig. 5e).

### CHIR99021 treatment blocks melanoma growth in xenografts and significantly increases survival of genetically engineered melanoma mice

**Fig. 6:**
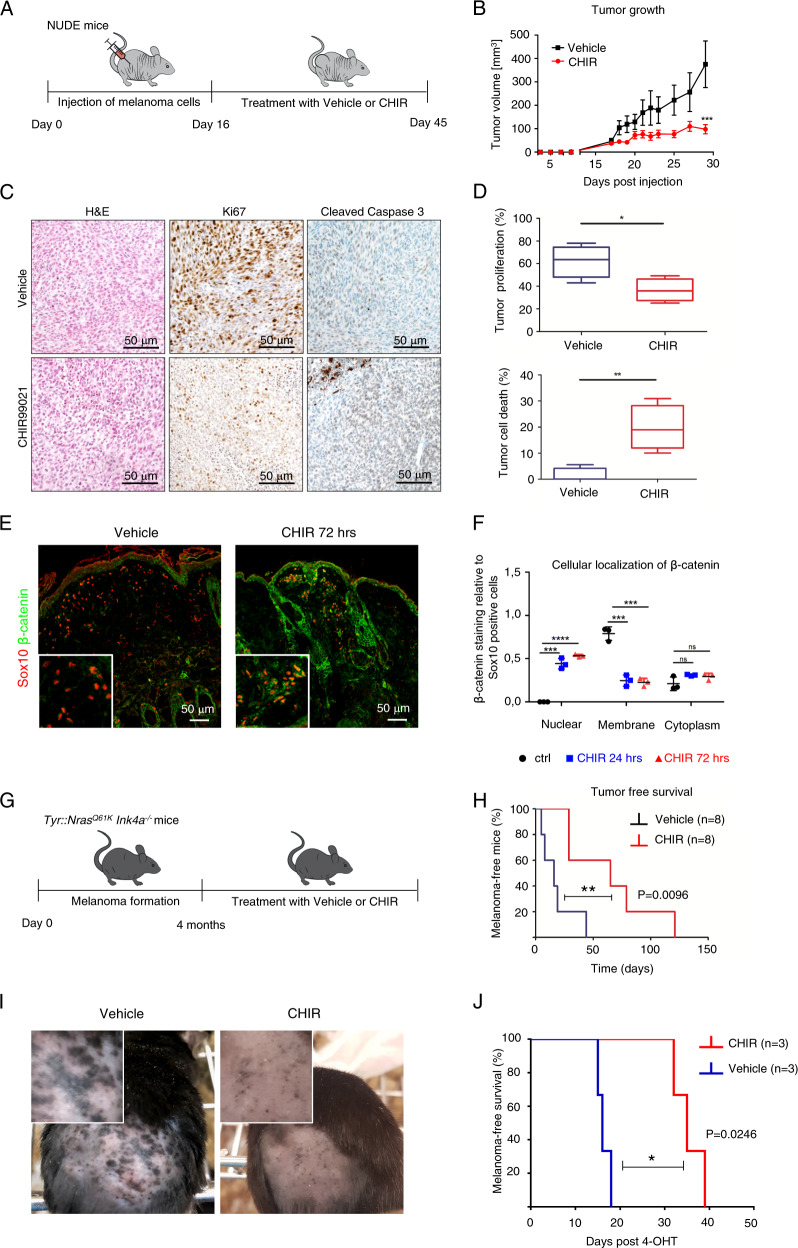
CHIR99021 treatment blocks tumor growth in xenografts and improves overall survival of genetic melanoma mouse models. **a** Experimental scheme of injection of the MAPK inhibitor resistant human melanoma cell culture M11 into both flanks of immunocompromised mice. Subsequent i.p. treatment with vehicle (Captisol) or CHIR99021 (*n* = 4/treatment group). **b** Tumor growth kinetics with (*n* = 4) and without (*n* = 4) CHIR99021 treatment in xenografts established from the MAPK inhibitor resistant human melanoma cell culture M11. **c** Representative histologic analysis (*n* = 8) of H&E, Ki67 and Cleaved Caspase 3 stainings of patient-derived xenografts treated with vehicle (Captisol) (top) or CHIR99021 (bottom). **d** Quantification of stainings of proliferative (top) and apoptotic cells (bottom) in M11-derived xenografts treated with either vehicle (Captisol) or CHIR99021. **e** Representative immunostaining of Sox10 (red) and β-catenin (green) of skin of 5-month-old mice from the *Tyr::Nras*^*Q61K*^*INK4a*^*−/−*^ melanoma mouse model treated with vehicle (Captisol) (left panel) or CHIR99021 (right panel) daily for 3 days in a row. Inserts show higher magnification of Sox10 and β-catenin immunostaining. **f** Quantification of subcellular localization of β-catenin of mice skin treated with either Captisol (vehicle) or CHIR99021 for 24 or 72 h (once every 24 h, 30 mg/kg). **g** Experimental scheme of treatment of 4–5-month-old-mice from the *Tyr::Nras*^*Q61K*^*INK4a*^*−/−*^ melanoma mouse model with either vehicle (Captisol) or CHIR99021 (30 mg/kg) (*n* = 8/treatment group). **h** Kaplan–Meier survival analysis of vehicle (Captisol) treated mice compared to CHIR99021 treated mice (*n* = 8/treatment group) from the *Tyr::Nras*^*Q61K*^*INK4a*^*−/−*^ melanoma mouse model. **i** Representative picture of two *Braf*^*V600E*^ *Pten*^*−/−*^ *Tyr::CreERT2* mice (6–10 weeks old) treated with vehicle (Captisol) (left) or with 30 mg/kg CHIR99021 (right) sacrificed at the same time. **j** Kaplan–Meier survival analysis of vehicle (Captisol) treated mice compared to CHIR99021 treated mice (*n* = 3/treatment group) from the *Braf*^*V600E*^ *Pten*^*−/−*^ *Tyr::CreERT2* melanoma mouse model. Data in **b** represent mean tumor volume ± s.e.m. Statistical significance was determined by log-rank (Mantel–Cox) test (**h**, **j**) or two-tailed Student’s *t* test (**b**, **d**, **f**). *P < 0.05, **P < 0.01, ***P < 0.001.

Next, we examined whether the profound effect of CHIR99021 on melanoma cells in vitro could also be observed in vivo. To this end, we injected Balb/c nude mice with a vemurafenib-resistant patient-derived melanoma culture (M111031). As soon as the first signs of tumor development were detected, we started to treat the mice with CHIR99021 (30 mg/kg) or vehicle control (Captisol) intraperitoneally (i.p.) (*n* = 6 for both groups) (Fig. 6a). The mice did not exhibit any signs of treatment-related side effects, as illustrated by the maintained body weight in both treated and untreated mice (Supplementary Fig. S9). As soon as the control group reached the tumor size that was defined as one of the termination criteria, all the mice were sacrificed and tumors were dissected. Consistent with our in vitro data, CHIR99021 efficiently blocked the growth of tumors in this xenograft model (mean tumor volume 375.7 mm^3^ in Captisol-treated mice versus 98.1 mm^3^ in CHIR99021-treated mice at day 29 post injection, *P* < 0.0001; Fig. 6b and Supplementary Fig. S10). Immunohistochemical analysis of the tumors revealed that CHIR99021 treatment significantly reduced cell proliferation as assessed by Ki67 staining. This was accompanied by increased levels of cleaved caspase-3, indicating an induction of apoptosis (Fig. 6c, d and Supplementary Fig. S11).

Next, we studied the effects of CHIR99021 in a genetically engineered mouse model (GEMM) of primary melanoma, the *Tyr::Nras*^*Q61K*^ *Ink4a*^*−*^^*/−*^ model. In this model, the *Nras p.Q61K* oncogene is expressed under the control of the tyrosinase promoter. Together with the *Ink4a* homozygous knockout, this model develops melanoma with 90% incidence at an age of 6 months [39]. First, we performed a pharmacodynamic analysis of CHIR99021 treatment. For this purpose, we injected 30 mg/kg CHIR99021 i.p. daily and euthanized the mice after 24 or 72 h. Immunohistochemical analysis of the skin revealed a clear reduction of Sox10 expression after 72 h and β-catenin levels in Sox10 positive cells were increased compared to mice treated with vehicle control (Fig. 6e, f). Next, we started a long-term treatment of *Tyr::Nras*^*Q61K*^ *Ink4a*^*−/−*^ mice that showed the first signs of tumor development. As in the xenograft experiment, mice were treated either with 30 mg/kg of CHIR99021 in Captisol, or with vehicle control (Captisol 15%). Treatment was daily for fourteen days in a row, followed by a maintenance treatment of once a week until reaching termination criteria (Fig. 6g). Captisol-treated mice rapidly progressed and showed signs of melanoma development, requiring euthanasia within 1–7 weeks after treatment start. However, CHIR99021 treatment significantly improved median overall survival from 2.3 weeks (Captisol) to 9.3 weeks (CHIR99021), after treatment start (Fig. 6h and Supplementary Fig. S12). Finally, we investigated whether the effect of CHIR99021 treatment in *Tyr::Nras*^*Q61K*^ *Ink4a*^*−*^^*/−*^ mice could be validated also in another GEMM of melanoma.

For this purpose we focused on *Braf*^*V600E*^ *Pten*^*−*^^*/−*^ *Tyr::CreERT2*, an inducible genetic melanoma mouse model. The inducible Cre recombinase under the Tyrosinase promoter is induced by 4-hydroxytamoxifen and drives expression of the *Braf*^*V600E*^ oncogene, the most common mutation found in human melanoma patients, combined with a homozygous loss of the tumor suppressor gene *Pten*. As observed in the *Tyr::Nras*^*Q61K*^ *Ink4a*^*−*^^*/−*^ mouse model, the overall survival in the *Braf*^*V600E*^ *Pten*^*−*^^*/−*^ *Tyr::CreERT2* was also prolonged significantly in the CHIR99021 treated group compared with the control group (Fig. 6i, j).

**Fig. 7:**
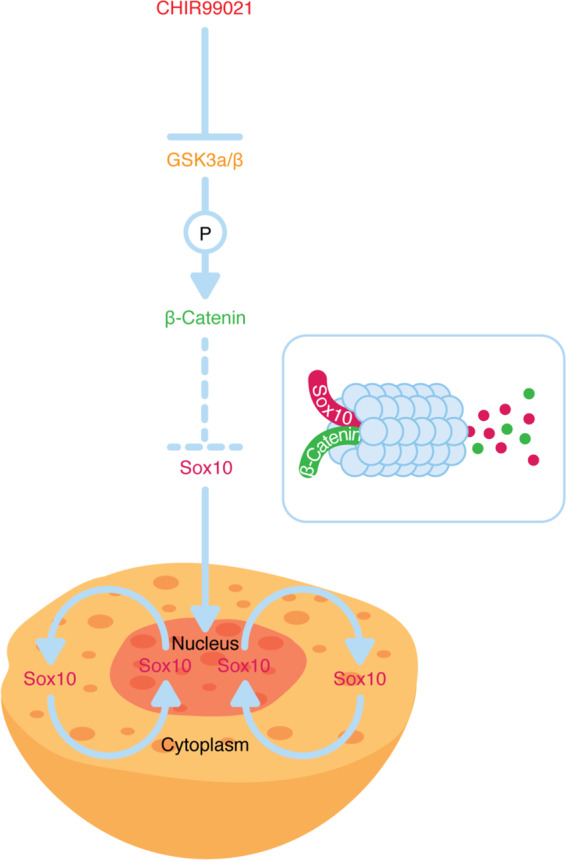
A schematic illustration of the effect of CHIR99021 on the level of SOX10 protein. CHIR99021 is a small chemical compound that competes with ATP for the binding site in GSK3α/β kinase and thereby inhibits GSK3α/β, leading to the activation of WNT signaling. SOX10 protein levels are reduced following pharmacologic, acute β-catenin stabilization, and this is mediated via proteasomal degradation.

Consistent with in vitro data, we demonstrate that temporal pharmacologic activation of WNT/β-catenin signaling by treatment with the GSK3α/β inhibitor CHIR99021 markedly decreases melanoma tumor growth by inhibiting proliferation and inducing apoptosis also in GEMM of melanoma (Fig. 7).

## Discussion

Although recent great improvements in therapies against metastatic melanoma have been achieved using targeted inhibitors against the MAPK signaling pathway or immune checkpoints, primary and secondary resistance against those approaches still pose a major clinical problem. By far, most patients suffering from metastatic melanoma will eventually succumb to their disease [1]. Finding new therapies that improve the survival of malignant melanoma patients is therefore still a highly unmet medical need. β-catenin is known to be dysregulated in many types of cancers, including melanoma [40]. Although β-catenin mutations have been detected in 23% of melanoma cell lines [29], this is a rare event in primary, uncultured melanomas (about 5%) [41–44]. The impact of this deregulation and the functional implications are very controversial and have not been fully clarified. Increased levels of β-catenin have been associated with a better outcome for melanoma patients [23]; whereas other publications found that the loss of β-catenin counteracts melanoma formation [17]. This shows that WNT/β-catenin signaling has a profound, context-dependent impact on melanoma formation. In order to analyze these contradictory assertions, we further investigated the role of canonical WNT signaling in SOX10 regulation in melanoma.

SOX10 is a transcription factor that is crucial in neural crest development and we have recently identified it as a novel promising candidate for melanoma treatment [12].

Interestingly, the targeted inhibitors of the MAPK signaling pathway, which are successfully being used in clinical routine, did not alter SOX10 levels in primary melanoma cultures in vitro, nor in vivo in human patient samples, suggesting that maintaining SOX10 levels might be a mechanism of later, acquired resistance. However, we have now identified a new mechanism to successfully suppress SOX10 pharmacologically in malignant melanoma. We have established a connection between SOX10 and the WNT/β-catenin signaling pathway by identifying β-catenin as a direct protein-protein interaction partner of SOX10 in an unbiased MS-based approach in melanoma. This interaction has functional consequences, since in contrast to the situation in neural crest development where activation of WNT signaling results in expression of SOX10 [12], activation of WNT signaling by inhibiting the negative regulator GSK3α/β pharmacologically using different compounds potently suppresses SOX10 in primary melanoma cultures. This reduces cell survival and invasion in vitro, as well as tumor formation in vivo. Suppression of SOX10 expression occurs at the protein, not at the mRNA level, and it is mediated by the proteasome, since proteasome inhibition rescues SOX10 expression. Importantly, the suppressive effect on SOX10 is β-catenin dependent, as shown by shRNA-mediated rescue experiments. Moreover, RNA-sequencing findings showed a large overlap in transcriptomic changes between shRNA-mediated SOX10 suppression and suppression induced by treatment with a GSK3α/β inhibitor. This observation further confirms a specific effect induced by WNT/β-catenin signaling, rather than an off-target effect mediated by other GSK3α/β substrates. Intriguingly, a study by Zhou et al. demonstrated that SOX10 acts as an oncogene and interacts with WNT/β-catenin signaling in hepatocellular carcinoma [45].

The role of WNT/β-catenin signaling in melanoma initiation and progression is very controversial despite extensive in vitro studies, in vivo modeling, and patient cohort studies. On the one hand and in line with our observations, there are several studies suggesting that activation of WNT/β-catenin might be a potential therapeutic strategy. It was shown to reduce melanoma cell proliferation in vitro by inducing genes involved in melanocyte differentiation and in line with that, nuclear β-catenin expression was lost during melanoma progression in patient samples [23]. In another study, pharmacologic inhibition of GSK3α/β was shown to reduce proliferation of melanoma cells and in vivo growth in a xenograft experiment [21]. Third, in a chemical-genetic screen riluzole was found to activate WNT/β-catenin signaling in melanoma cells, to promote pigmentation and to decrease proliferation [46]. And lastly, secreted WNT5A (as opposed to WNT3A) has been shown to negatively regulate WNT/β-catenin signaling in several cancer types, including in a subgroup of melanoma patients exhibiting acquired resistance to BRAF-inhibition [19]. Reducing WNT5A by siRNA in this model activated canonical WNT signaling and provoked cell death, counteracting melanoma formation. However, none of those studies related activation of WNT/β-catenin to suppression of SOX10 protein levels and connected this event to the observed phenotypic changes.

On the other hand, some studies presented apparently contrary results and made observations that activation of WNT/β-catenin promotes melanomagenesis and metastatic spread. Damsky et al. utilized the *Braf*^*V600E*^*/Pten*^−*/−*^ GEMM to study the effect of β-catenin alterations on melanoma initiation and metastasis [17]. β-catenin knock out abrogated formation of melanoma in this model, whereas introduction of an exon 3-deleted, pathologically stabilized β-catenin mutant mimicking constitutively activated WNT signaling increased metastasis formation. Sox10 expression was assessed at the mRNA level only in these mouse models, and no differences were observed, in line with our observations that this regulation happens at the protein level. However, this mouse model represents only a minority of melanoma patients, since exon-3 mutations are exceedingly rare in primary and metastatic cutaneous or uveal melanomas, as has been published and as shown by our analysis of the TCGA database [42, 47, 48]. No conclusions can be drawn from those observations with respect to the potential therapeutic benefit of activating WNT/β-catenin signaling in melanomas with wild-type β-catenin. Indeed, we have also observed that inhibiting GSK3α/β in melanoma cell lines carrying a classical exon-3 mutation (β-catenin p.S37F [29]) in the GSK3α/β consensus site did not have any effect on SOX10 protein levels. Presumably, because the cells have bypassed the SOX10 suppressing effect of activated WNT/β-catenin signaling and adapted to the constantly elevated signaling levels in order to maintain SOX10. From those findings, one might also conclude that the small subset of melanoma patients harboring a stabilizing exon-3 mutation may not derive benefit from treatment with GSK3α/β inhibitors.

Another study showed that WNT5A can exert a dichotomous role in melanoma [49]. On the one hand it signals through the ROR2/Fzd2/5 receptor complex to degrade β-catenin, on the other hand it can also signal through the LRP6/Fzd4 receptor complex to stabilize β-catenin via ARF6. Interestingly, both mechanisms appeared to increase invasion. However, experimental proof that the latter mechanism is functionally dependent on β-catenin is lacking and it is therefore also conceivable that WNT5A signals through ARF6 and unknown intermediates to regulate invasion independent from β-catenin and that stabilization of β-catenin in this context is a bystander rather than e driver effect (reviewed in [18]). Our rescue and the RNA-sequencing experiments on the other hand demonstrate that the impairment of melanoma cell survival provoked by SOX10 suppression upon GSK3α/β inhibition is dependent on β-catenin, suggesting a direct involvement of canonical WNT/β-catenin signaling.

Taken together, we have identified a SOX10 suppressing function of activating WNT/β-catenin signaling pharmacologically, by inhibiting its negative regulator GSK3α/β. This impacted significantly on survival of melanoma cells. Importantly, this approach was also efficient in primary melanoma cultures, which had been established from patients with an acquired resistance to TKI treatment. Our findings presented in this work, and a previous study from the Moon lab [23] suggest that pharmacological activation of WNT/β-catenin signaling in patients with malignant melanoma has therapeutic potential and should next be tested in early phase clinical trials.

## Methods

### Reagents

CHIR99021 (S1263, Selleckchem, distributed by LuBioScience, Zurich, Switzerland) was used at different concentrations. LY2090314 (S7063, Selleckchem) was used at 100 nM. AZD1080 (S7145, Selleckchem) was used at 10 µM. Vemurafenib (PLX4032, RG7204), (S1267, Selleckchem) was used at 1 μM. Dabrafenib (S2807, Sellechchem) was used at 1 μM. Selumetinib (AZD6244), (S1008, Selleckchem) was used at 1 μM MG-132, (S2619, Selleckchem) was used at 20 μM.

### Cell culture

A panel of different patient-derived melanoma cultures was obtained from the laboratory of Prof. M. Levesque from Dermatology, USZ (cf Table 1). Two more cell lines from the laboratory of L. Sommer, Anatomy, UZH were obtained; these cell lines harbor a mutation in β-catenin (pS37F). Cells were cultured in RPMI-1640 medium (L0998-500, Dominique Dutscher) enriched with 10% FCS (S181B-500, Dominique Dutscher), L-Glutamine, and Na-Pyruvat (11360-039, Gibco) (1% each).

### Proliferation, invasion, colony formation, and cell viability assays

#### **Colony formation assay**

3000 cells/well were plated in a six-well plate. Medium was changed every other day until the control was confluent. The cells were subsequently fixed for 10 min in 4% paraformaldehyde (109444 U-58, Kantonsapotheke Zürich, Switzerland, 4% in PBS) and stained with 0.5% of Crystal Violet solution (2500 ml of ethanol 96%, 25 g of Crystal Violet, 40 g NaCl, 2250 ml water, 250 ml paraformaldehyde 37%).

#### Cell viability assay

2000 cells/well were plated in a 96 well plate. After 24 h of incubation, cells were cultured in triplicates in the presence of either vemurafenib or CHIR99021 at serial dilution concentrations of the drug. After 72 h the medium was removed and 100 μL of resazurin (R7017, Sigma), 0.5 mg/ml was added for 1–4 h and incubated at 37 °C. The absorbance at 570 nm was measured using a Spark multimode microplate reader (TECAN, Männedorf, Switzerland).

#### Invasion assay

Cells were seeded 48 h prior to the assay at 60–80% density. The cells were starved (3% FCS) for 48 h. Inserts (354480, Corning Biocoat Matrigel Invasion Chamber for invasion assay) were placed into 24 well chambers (containing 800 µl of starving medium) and filled with 500 µl starving medium and left for rehydration for 2 h. The medium from the insert was removed and between 350 and 500 µl of a 1 × 10^5^ cells/ml cell suspension was added. 800 µl of complete medium was added to the well. The plate was then incubated for 22 h. The invasion potential of the cells was examined by staining the remaining cells in the insert with 1:1000 Hoechst 33342 Tri-Hydrochloride, Trihydrated (H1399, Molecular probes by life technologies) in PBS.

#### shRNA lentivirus production and infection

The following shRNAs were used for long-term lentiviral knockdowns: SOX10 (TRCN0000018984, Sigma-Aldrich), MITF (TRCN0000329793, Sigma-Aldrich), β-catenin (TRCN0000314920, The RNA Consortium (TRC Project, Broad Institute). Nssh: mission shRNA control vector SHC002 – caacaagatgaagagcaccaa; sh20: pLKO.1 shCTNNB1_TRCN0000314920 – gcttggaatgagactgctgat; sh21: pLKO.1 shCTNNB1_TRCN0000314921 – tctaacctcacttgcaataat; sh77: pLKO.1 shCTNNB1_TRCN0000314977 – gggagtggtttaggctatttg. Lentivirus production and subsequent infection of melanoma cell lines, was performed following Addgenes’ protocol. In brief, melanoma cultures were plated and infected the following day with virus. After 24 h the virus-containing-medium was removed, and after 48 h puromycin (sc-108071A, Santa Cruz) was added (1 µg/ml) to select for the cells that stably integrated the shRNA.

### RNA extraction, reverse transcription, and quantitative PCR

Whole RNA was isolated and DNAse treated from the melanoma cultures using the RNeasy Mini Kit (74106, Qiagen) according to the manufacturers’ protocol.

One microgram RNA was reverse transcribed using GoScript Reverse Transcriptase (A501C, Promega) and oligo(dT) 15 Primer (C110A, Promega). Quantitative real time PCR was performed using the Rotor-Gene SYBR Green PCR kit (204076, Qiagen) on a Rotor-Gene Q instrument (SN: R 030962, Qiagen). RNA levels were normalized to the housekeeping gene *GAPDH*. The following primers were used: huGAPDH fw 5′ ACCACAGTCCATGCCATCAC; huGAPDH rv 5′ TCCACCACCCTGTTGCTGTA; huSOX10 fw 5′ CCTCACAGATCGCCTACACC; huSOX10 rv 5′ CATATAGGAGAAGGCCGAGTAGA.

### RNA sequencing and analysis

For each of the melanoma cultures (M980513, M000921, and M111031) there were two different conditions, performed in biological triplicates, each: (i) control (DMSO), (ii) 6 μM CHIR99021 for 24 h. The RNA extracted from the melanoma cultures was sent to Functional Genomic Center Zurich for RNA sequencing. Datasets are available under GEO accession number 146415.

### Bioinformatics

The Illumina reads recorded in fastq files, have been processed with trimmomatic (v 0.35), then aligned to the human genome (GRCh38) using Star [50] (v 2.4.2a), then processed with samtools. The reads for each gene have been counted using featureCounts (v1.5.0) from the subread package [51]. The count table has been processed statistically, looking for differential expression using generalized linear model approach with edgeR Bioconductor [52] packages with appropriate contrasts between groups. The differences between the groups have been studied using heatmaps (pheatmap) [53]. Annotated WNT and SOX10 transcriptional target gene lists were obtained from Metacore (https://portal.genego.com/).

### Western blotting

Treated cells were washed and collected in ice-cold PBS. The proteins were extracted from the cell pellet using TNN lysis buffer (Tris pH 7.5 50 mM, NaCl 250 mM, EDTA 5 mM, NP-40 0.5%, NaF 50 mM, EGTA 0.5 mM), containing complete ULTRA protease inhibitor (05892970001, Roche) and PhosSTOP (4906837001, Roche). After 15 min of incubation on ice, cell lysates were collected for 13 min at 14,000 rpm at 4 °C. The protein-containing supernatant was used immediately. We quantified the extracted proteins by Pierce BCA assay according to the manufacturers’ instructions (23227, ThermoFisher Scientific). 30 µg of whole cell protein lysates were loaded on precast Tris–HCl gels, 4–20% (456–8093, Bio-Rad). The gels were run for 10 min at 80 V and then for 1 h at 160 V. The gels were blotted onto PVDF membranes (170–4156, Bio-Rad) using Trans-Blot Turbo Transfer System (690BR013492, BIO-RAD) and blocked for 1 h in TBS containing 0.1% Tween (TBST) and 5% milk. The following antibodies were used for detecting protein: Anti-SOX10 BC34, (ACI3099, BioCare Medical), 1:1000 in TBST and 5% BSA, anti-β-catenin (E-5), (sc-7963, Santa Cruz), 1:100 in TBST and 5% milk, anti-Phospho-p44/42 MAPK (Erk1/2) (Thr202/Tyr204) (9101S, cell signaling), 1:1000 in TBST and 5% BSA, anti-p44/42 MAPK (Erk1/2) (L34F12) (4696S, cell signaling), 1:1000 in TBST and 5% BSA, anti-MITF monoclonal antibody (C5), (MA5-14146, Thermo Fisher SCIENTIFIC), 1:50 in TBST and 5% milk. Anti-GAPDH Loading Control Monoclonal Antibody (GA1R), (MA5-15738, Thermo Fisher SCIENTIFIC), 1:1000 in TBST and 5% BSA. Anti-β-Actin, clone AC-74, (A5316, Sigma-Aldrich), 1:10,000 in TBST and 5% milk. The PVDF membranes were incubated with antibodies overnight at 4 °C. After three washings with TBST the membranes were incubated with HRP-coupled secondary antibodies for 1 h at room temperature (1:2500). The following antibodies were used: HRP Goat anti-mouse IgG, clone Poly4053, (405306, Biolegend), HRP anti-rabbit IgG, clone 6B9G9, (410406, Biolegend). The signal was detected using Fusion Fx VILBER LOURMAT (12-200168).

### Co-immunoprecipitation

For co-IP experiments, HEK293T/17 cells were transfected with plasmids pcDNA3.1 (Addgene), flag-β-catenin (Addgene), and/or HA-SOX10 (Invitrogen), as indicated in Fig. 2b, using Lipofectamine 2000 (Thermo Fisher Scientific, Illkirch, France) according to the manufacturer’s protocol. 24 h after transfection, cells were pelleted and lysed in IP buffer (50 mM Tris pH 8.0, 150 mM NaCl, 1 mM EDTA, 1% Triton X100), containing 0.4 µl/ml Benzonase (E1014-5KU, Sigma), 20 µM MG-132 (S2619, Selleckchem), and PhostSTOP and cOmplete (Roche), as described above. Protein concentration was then measured by Pierce BCA assay, as described above. IP was peformed using Dynabeads® Protein G (Novex, Life Technologies), according to the manufacturer’s protocol. Briefly, 50 µl of beads were coupled to 6 µg of anti-HA antibody (H6908, Sigma) for 10 min at room temperature. After washing, 2000 µg of cell lysate in IP buffer was added and incubated for 2 h at 4 °C on a rotating wheel. After washing, immunoprecipitates were eluted in elution buffer (50 mM glycine pH 2.8) and NuPAGE® Sample buffer (Thermo Fisher) by heating at 70 °C for 10 min. Eluates were then loaded for SDS-PAGE and blotted as described above.

### Histochemistry, immunohistochemistry, and immunofluorescence

Mouse skin samples were fixed in 4% phosphate-buffered formaldehyde solution (P078.3, Roth) for 2 h and subsequently embedded in paraffin (MEDITE PURE Paraffin, 40-0020-00, MEDITE). Sections of 5 µm thickness were deparaffinized and rehydrated using the Automatic Staining System AS-2 (SN: 180.001.1015.119, PATHISTO) and antigen retrieval was performed in citrate buffer (pH 6.0) for 25 min at 110 °C using Decloaking Chamber (SN: DG12-220-0134, BIOCARE MEDICAL).

### Hematoxylin and eosin staining

Slides were first deparaffinized 10 min for two times in Histoclear (D1620333, Chemie Brunschwig), then rehydrated for 1 min each in 100, 96, 80, 70% EtOH (179-VL03K-/1, Thommen Furler AG) and ddH2O following 7 min in filtered hematoxylin, three times 10 s in ddH_2_O, 30 s in Scott Water (8 mM MgSO_4_ × 7H_2_O (M2643-500G, Sigma-Aldrich), 24 mM NaHCO_3_ (S5761, Sigma-Aldrich) and 1.5 min in Eosin (0.2%, 41-6660-00, Medite) containing three drops of CH_3_COOH. Subsequently slides were placed two times 10 s each in 50, 70, 80, 95, and 100% EtOH and finally two times 10 min in Xylol (253-VL03K, Thommen Furler AG). Then they were mounted using Eukit Quick hardening mounting medium (03989, Fluka).

### Immunohistochemistry

After antigen retrieval, sections were incubated with permeabilization buffer (TBS and 0.5% Triton X-100, 93426, Sigma) for 10 min and washed three times with PBS. Blocking and antibody incubation were performed as described in the manufacturer’s protocol (Vectastain ABC Kit, PK-4000, Vector Laboratories). Signal detection was performed using the DAB Substrate Kit (ab64238, Abcam) according to the manufacturer’s protocol.

### Immunofluorescence

Sections were incubated with permeabilization buffer (TBS and 0.5% Triton X-100, 93426, Sigma) for 10 min, washed three times with PBS and subsequently incubated in blocking buffer (PBS, 5% horse serum, and 0.1% Triton X-100) for 45 min. The slides were incubated overnight at 4 °C with the following primary antibodies: anti-SOX10 (D5V9L), (89356, Cell Signaling), 1:100 in PBS, phospho-ERK1/ERK2 (Thr185, Tyr187), (700012, 15H10L7), Thermofischer Scientific 1:100 in PBS, purified mouse anti-β-catenin (6110154, BD Transduction Laboratories), 1:200 in PBS, pan Melanoma-Cocktail 2 (MART-1 and Tyrosinase), (CM 178 A, BIOCARE MEDICAL), 1:150 in PBS, Ki67: (15580, Abcam) 1:200 in PBS. After washing three times for 10 min, the following secondary antibodies were applied: 1:250 in PBS for 1 h: Alexa Fluor 647 AffiniPure Donkey Anti-Mouse IgG (H+L), (715-605-151, Jackson ImmunoResearch LABORATORIES, INC.) Cy^TM^3 AffiniPure Goat Anti-Rabbit IgG (H+L), (111-165-003, Jackson ImmunoResearch, LABORATORIES, INC.) After three washing steps in PBS, melanoma cultures were optionally incubated with Phalloidin control DyLight488 (21833, Invitrogen) 1:250 for 20 min at room temperature. After three washing steps, the nuclei were stained and mounted with DAKO Fluorescent Mounting Medium (S3023, DAKO) and Hoechst 33342 Tri-Hydrochloride, Trihydrated (H1399, Molecular probes by life technologies), 1:1000 for 7 min. The images were captured with Leica DMI6000 B or Leica SP8 inverse confocal microscope and analyzed using the LAS AF or Leica LAS X software. Melanoma patient biopsies were analyzed before and after clinical treatment with immunohistochemistry. Stainings were conducted as described above. TMA paraffin sections used in this study were obtained from the Institute of Pathology and Molecular Pathology, University Hospital Zurich.

### Immunocytochemistry assay

5000 cells were plated into μ-Slide 8 well ibiTreat (80826, ibidi). After treatment, the medium was removed and the cells were fixed using 4% PFA for 10 min. After three washing steps, cells were permeabilized for 5 min. The staining procedure was carried out as described for immunofluorescence.

### Animal studies

All animal experiments were performed in accordance with Swiss law and have been approved by the veterinary authorities of Zurich.

### Xenografts

1 × 10^6^ cells (M111031) were injected subcutaneously in both flanks of immunocompromised mice (CanN.Cg-Foxn1<nu>/Crl (BALB/c-nude), Charles River). When first signs of tumors were appearing, mice were injected i.p. with 30 mg/kg of CHIR99021 (S2924, Selleckchem), diluted in captisol (RC-0C7-020, captisol) or captisol alone (15%) until the tumor reached the size of 1 cm^3^.

### Melanoma mouse model experiments

Mice from the *Tyr::Nras*^*Q61K*^ *Ink4a*^*−/−*^ model were injected i.p. for 14 days in a row with CHIR99021 (30 mg/kg) followed by weekly injections. Another group of mice served as control and was injected with captisol (15%) at the same intervals as the treatment group. The mice were euthanized when they reached one of the termination criteria, and skin and organs were embedded in paraffin for immunohistochemistry analysis. Another group of the *Tyr::Nras*^*Q61K*^ *Ink4a*^*−/−*^ mice was injected i.p. with CHIR99021 (30 mg/kg) and euthanized 24 or 72 h after injection. Skin and organs were analyzed by immunohistochemistry. Mice from *Braf*^*V600E*^ *Pten*^*−/−*^ *Tyr::CreERT2* model (6–10-week old) were shaved on the back skin and 4-hydroxytamoxifen in DMSO was applied topically on 3 consecutive days (25 mg/ml). 1 week after the first application, mice were treated i.p. daily with CHIR99021 (30 mg/kg) for 2 consecutive weeks, followed by weekly treatment until termination criteria were reached. Skin and organs were dissected and embedded in paraffin for immunohistochemistry analysis.

### CovalX experiment

#### nLc Orbitrap mass spectrometry to identify SOX10-interacting proteins

To identify novel SOX10-interacting proteins, a M010817 WCE was analyzed by nLc Orbitrap MS at CovalX (CovalX AG, Zürich, Switzerland). M010817 were lysed in NE buffer (50 mM Tris pH 7.5, 150 mM KCl, 0.2 mM EDTA, 5 mM MgCl_2_, 20% Glycerol, 0.5 mM DTT) and then sent to CovalX for further analysis, along with purified SOX10 protein in a buffer of 25 mM Tris pH 7.3, 100 mM glycine, 10% glycerol, obtained from Creative Biomart (Creative Biomart Inc, Shirley, NY, USA). Procedure at CovalX were as follows:

#### Dialysis

In a first step, the protein extract and SOX10 were subjected to dialysis. 50 μL of WCE, and 50 μL of SOX10 were pipetted in the dialysis device (Slide-A-Lyzer™, Thermofisher), and dialyzed against 1 ml of PBS, at 4 °C. After 2 h of incubation, the buffer was changed with 1 ml of fresh PBS solution for an additional overnight incubation at 4 °C.

#### Mixing samples and cross-link experiments

Three different ratios of WCE/SOX10 were prepared for the interaction study: 1/1, 1/5, and 1/10. Mixtures prepared were submitted to crosslinking using 0.5 μl of DSS d0/d12 (2 mg/ml, N,N-Dimethylformamide) for the ratio 1/1, 2.4 μL of DSS d0/d12 for the ratio 1/5 and and 4.8 μL of DSS d0/d12 for the ratio 1/10. The cross-link samples were incubated for 3 h at room temperature. After incubation, 2 μl of a solution of ammonium bicarbonate (500 mM) was added to the solutions in order to stop the cross-linking reactions. After 1 h of incubation time, the solution was evaporated to dryness and suspended in 20 μL of 1X bromophenol blue.

#### Trypsin proteolysis

20 μL of each solution was separated by SDS-PAGE and analyzed by Coomassie blue staining. The protein band was cut, and proteins reduced with dithiothreitol, alkylated with iodoacetamide, and finally in-gel digested with trypsin (overnight, 37 °C). Peptides were extracted from the gel, evaporated to dryness, and suspended in H_2_O/formic acid 0.1% to a final concentration of 0.1 μg/μL for ratio 1/5 and 1/10 and 0.05 μg/μl for ratio 1:1. 10 μL of each solution was injected into the Orbitrap for nLC MS analysis with one injection for Sequest HT search engine and another injection for Xquest search engine.

#### Instrumentation

Measurements were performed using an LTQ orbitrap mass spectrometer (ThermoFisher) coupled with a nano LC chromatography (Ultimate 3000, Dionex), equipped with C18 column (75 μm ID × 15 cm nanoViper C18, 3 μm, 100 Å – Acclaim^®^ PepMap100).

### Statistics

Data are presented as the mean ± s.d. of independent experiments, unless otherwise specified. Statistical analyses were performed with GraphPad Prism 7.0 (GraphPad Software) using 2-way ANOVA for multigroup comparisons or two-tailed Student’s *t* test for 2-group comparison, unless otherwise specified in the figure legends. For survival analysis a Log-Rank (Mantel–Cox) test was performed to determine statistical significance. A *P* value of <0.05 was considered to be significant.

### Study approval

All patient material was provided by consenting melanoma patients from the University of Zurich Hospital, according to local IRB approval (EK.647/800, and KEK-ZH.Nr.2014-0425).

## Supplementary information

Supplementary Figure legends

Supplementary Figures
