# Supplementary material for: Temporal activation of WNT/β-catenin signaling is sufficient to inhibit SOX10 expression and block melanoma growth
Source: Oncogene. 2020 Apr 1;39(20):4132–54. doi: 10.1038/s41388-020-1267-7 (PMC8076051; doi:10.1038/s41388-020-1267-7)
Supplement: Supplementary file 1 — Supplementary Figure legends [file 41388_2020_1267_MOESM1_ESM.docx]

# Supplementary Figures Legends

**Figure S1. Vemurafenib only inhibits growth of some human melanoma cell cultures, whereas others are resistant.**

(**A**) Selected panel of human melanoma cultures and their characteristics. (**B**) Vemurafenib dose response curve for MAPK inhibitor sensitive (M98) and resistant (M11, M12) human melanoma cell cultures for 72 hours. Drug concentration is indicated in a logarithmic scale (*n*=3). *n* indicates the number of independent experiments performed.

**Figure S2. A schematic illustration of interacting domains on β-catenin and SOX10.**

**Figure S3. GSK3 α/β inhibitor LY2090314 also inhibits cell proliferation.**

**(A)** Representative pictures of the MAPK inhibitor sensitive (M01 and M98) and resistant melanoma patient-derived cell culture M12 in presence of DMSO (left panel) or LY2090314 (100nM) for 24 hours (right panel). **(B)** Colony formation potential in presence of 100nM of LY2090314 in MAPK inhibitor sensitive (M98, left panel) and a resistant (M12, right panel) human melanoma cell cultures is depicted (*n*=3). *n* indicates the number of independent experiments performed.

**Figure S4. GSK3 α/β inhibitor AZD1080 also inhibits cell proliferation and downregulates SOX10 expression levels.**

**(A)** Representative pictures of the MAPK inhibitor sensitive (M98) and resistant melanoma patient-derived cell cultures (M11, M12) in presence of DMSO (left panel) or AZD1080 (10µM) for 24 hours (right panel). **(B)** Colony formation potential in presence of 10µM of AZD1080 in MAPK inhibitor sensitive (M98, left panel) and a resistant (M12, right panel) human melanoma cell cultures is depicted (*n*=3). **(C)** Representative western blot for the indicated proteins in the MAPK inhibitor sensitive (M98, M00) and resistant (M11, M12) melanoma patient-derived cell cultures in presence or absence of AZD1080 (24 hours at 10µM). β-actin is used as loading control (*n*=3). *n* indicates the number of independent experiments performed.

**Figure S5. Endogenous expression levels of β-catenin and SOX10.**

Representative western blot of indicated proteins of a panel of human melanoma cell cultures, harboring mutations described in table 1. Resistance towards MAPK inhibitors are indicated in table 1. GAPDH was used as loading control (*n*=3). *n* indicates the number of independent experiments performed.

**Figure S6. Diagram of β-catenin mutations in melanoma patients.**

Colors of circles correspond to mutation types. Color code and corresponding mutation types are as follows: In green missense mutations, in black truncating mutations: Nonsense, nonstop, frameshift deletion, frameshift insertion and splice site, in brown inframe mutations: inframe deletion, inframe insertion.

**Figure S7. Endogenous expression levels of SOX10 and β-catenin are rescued after 24 to 48 hours of CHIR99021 withdrawal.**

Representative western blot of the indicated proteins showing the recovery of endogenous SOX10 and β-catenin expression levels after 72 hours of CHIR99021 (at 6µM) treatment and subsequent change to ctrl medium (24 hours or 48 hours) in MAPK inhibitor sensitive (M98) or resistant (M11) human melanoma cell cultures. GAPDH was used as loading control (n=3). *n* indicates the number of independent experiments performed.

**Figure S8. mRNA levels of SOX10 remain unchanged upon CHIR99021 treatment in MAPK inhibitor sensitive and resistant melanoma patient-derived cell cultures .**

**(A)** RNA levels in MAPK inhibitor sensitive (M00) human melanoma cell culture (ΔCt to *GAPDH* expression) after 4, 8 and 12 hours of CHIR99021 (6µM) treatment are shown compared to DMSO (ctrl) (*n*=3). **(B)** RNA levels in MAPK inhibitor resistant (M11) human melanoma cell culture (ΔCt to *GAPDH* expression) after 4, 8 and 12 hours of CHIR99021 (6µM) treatment are shown compared to DMSO (ctrl) (*n*=3). Data represent mean +/- s.d. Statistical significance was determined by unpaired, two-tailed Student’s t-test. * P < 0.05, **P < 0.01, ***P < 0.001. In each panel, *n* indicates the number of independent experiments performed.

**Figure S9. Confirmation of SOX10 downregulation upon CHIR99021 treatment.**

Representative western blot for the indicated protein demonstrating the downregulation of SOX10 upon CHIR99021 (24 hours, 6µM) in M98 and M00 (MAPK inhibitor sensitive) and M11 (resistant) melanoma cell cultures. GAPDH was used as loading control.

**Figure S10. Body weight of nude mice remains stable upon CHIR99021 treatment.**

Body weight was maintained in treated (CHIR99021, 30mg/kg) and untreated (vehicle) mice, indicating no treatment-related side effects.

**Figure S11. Whole scans of patient-derived xenografts treated (CHIR99021) or untreated (vehicle) analysis.**

Representative histologic analysis (n=8) of H&E (top panel), Ki67 (middle panel) and Cleaved Caspase 3 (bottom panel) stainings of the MAPK inhibitor resistant culture M11-derived xenografts treated with vehicle (Captisol in the left panel) or CHIR99021 (in the right panel). Scale bars: 100µm.

**Figure S12. Treatment with CHIR99021 delays significantly melanoma formation in *Tyr::Nras^Q61K^INK4a^-/-^* mice.**

Representative picture of two 7 months old *Tyr::Nras^Q61K^INK4a^-/-^* mice treated with vehicle (Captisol) (left) or with 30mg/kg CHIR99021 (right) sacrificed at the same time. White arrows indicate melanoma formation.
